# Meta-analysis of DNA methylation aging signatures in 17 human tissues

**DOI:** 10.1038/s43587-026-01164-5

**Published:** 2026-06-26

**Authors:** Macsue Jacques, Kirsten Seale, Sarah Voisin, Anna Lysenko, Robin Grolaux, Bernadette Jones-Freeman, Severine Lamon, Mandhri Abeysooriya, Itamar Levinger, Carlie Bauer, Adam P. Sharples, Aino Heikkinen, Elina Sillanpaa, Miina Ollikainen, Cassandra Smith, James R. Broatch, Navabeh Zarekookandeh, Linn Gillberg, Ida Blom, Jesse R. Poganik, Mahdi Moqri, Vadim N. Gladyshev, Matthew Taper, Cassandra Malecki, Sean Lal, Nathalie Saurat, Steve Horvath, Andrew Teschendorff, Nir Eynon

**Affiliations:** 1 Australian Regenerative Medicine Institute, Monash University, Melbourne, Victoria Australia; 2 TruDiagnostics, Lexington, KY USA; 3 Institute for Health and Sport, Victoria University, Footscray, Victoria Australia; 4 Novo Nordisk, Copenhagen, Denmark; 5 Institute for Physical Activity and Nutrition, School of Exercise and Nutrition Sciences, Deakin University, Geelong, Victoria Australia; 6 Australian Institute for Musculoskeletal Science, Victoria University and Western Health, Melbourne, Victoria Australia; 7 Institute for Physical Performance, Norwegian School of Sport Sciences, Oslo, Norway; 8 Minerva Foundation Institute for Medical Research, Helsinki, Finland; 9 Institute for Molecular Medicine Finland, HiLIFE, University of Helsinki, Helsinki, Finland; 10 Faculty of Sport and Health Sciences, University of Jyväskylä, Jyväskylä, Finland; 11 Wellbeing Services County of Central Finland, Jyväskylä, Finland; 12 Nutrition & Health Innovation Research Institute, School of Medical and Health Sciences, Edith Cowan University, Perth, Western Australia Australia; 13 Medical School, The University of Western Australia, Perth, Western Australia Australia; 14 Department of Biomedical Sciences, University of Copenhagen, Copenhagen, Denmark; 15 Department of Biomedical Sciences, Faculty of Health and Medical Sciences, University of Copenhagen, Copenhagen, Denmark; 16 Division of Genetics, Department of Medicine, Brigham and Women’s Hospital, Harvard Medical School, Boston, MA USA; 17 Faculty of Medicine and Health, The University of Sydney and Royal Prince Alfred Hospital, Sydney, New South Wales Australia; 18 The Baird Institute for Applied Lung and Heart Research, Sydney, New South Wales Australia; 19 Altos Labs, Cambridge, UK; 20 Shanghai Institute of Nutrition and Health, Chinese Academy of Sciences, University of Chinese Academy of Sciences, Shanghai, China

**Keywords:** DNA methylation, Predictive markers, Ageing

## Abstract

Epigenetic changes, in particular DNA methylation, accumulate with age across different tissues, but whether these changes follow consistent patterns across different organs remains poorly understood. Here we show, through a meta-analysis of more than 15,000 human methylation profiles spanning 17 tissues, that aging produces both conserved and tissue-specific epigenetic signatures. We identify systemic shifts in methylation levels, increases in methylation variability, and growing molecular disorder across tissues. Network analysis revealed tightly connected gene clusters that are not modified by beneficial interventions, alongside a more modifiable cluster linked to NAD^+^ metabolism, supporting NAD^+^ as a potential therapeutic target in aging. A gene encoding a cell-adhesion protein, PCDHGA1, emerged as a conserved hub across tissues, implicating cell-to-cell communication pathways in aging across multiple organs. Our methylation atlas therefore provides a resource for dissecting the molecular basis of human aging and for identifying potential biomarkers and translational therapies.

## Abstract (web summary)

Jacques and colleagues present a cross-tissue meta-analysis of DNA methylation aging, revealing conserved aging signatures and modifiable gene clusters.

## Main

Aging is a universal yet profoundly individualized process. While chronological age progresses at a steady pace, its biological impact differs substantially among and within individuals, leading to varying risks of disease, functional decline and mortality^1,2^. These differences stem from cumulative molecular changes, with epigenetic modifications, particularly DNA methylation (DNAm) (the addition of methyl groups to cytosine bases), among the most reliable indicators of biological aging trajectories^1–3^.

Age-related DNAm changes fall into two main categories. Differentially methylated positions (DMPs) are sites exhibiting consistent directional changes across individuals with age, either gaining or losing methylation, and have been widely studied as potential biomarkers of biological aging^3–8^. Variably methylated positions (VMPs) instead show increased inter-individual variability with aging methylation levels among individuals, and this variability tends to increase with age, reflecting stochastic influences such as environmental exposures and genetic factors rather than uniform directional change^4,5,8,9^. A single CpG site can exhibit both properties simultaneously, showing a consistent directional shift across the population (DMP) while also becoming increasingly variable between individuals (VMP).^5,7^.

A third lens, Shannon entropy, captures the overall disorder of DNAm across the genome at the level of individual samples. Borrowed from information theory, entropy is highest when methylation at a given site is close to 50%, meaning the signal is maximally uncertain, and lowest when sites are fully methylated or unmethylated. As aging progresses, methylation patterns become less precise and more disordered, and entropy increases accordingly. Importantly, entropy integrates signals from both DMPs and VMPs, making it a complementary, system-wide measure of epigenetic aging that neither metric captures alone^4,9,10^.

Despite advancements in the field, most epigenetic aging research has primarily focused on blood samples, with few specific tissue investigations available, resulting in a limited body of work in the field^5,11–14^. As a result, fundamental questions remain unresolved: to what extent is the aging methylome shared across tissues, and where does it diverge? Are conserved methylation signatures reflective of systemic aging, or are they tissue-restricted phenomena? Understanding these dimensions is critical, as each tissue exhibits unique functional roles, cell-type compositions and regenerative capacities that shape distinct epigenetic aging trajectories. Identifying conserved patterns would also support the use of blood as a minimally invasive proxy to infer biological age in less accessible organs such as brain, heart or muscle.

To address these gaps, we assembled a comprehensive atlas of DNAm across 17 human tissues throughout the adult lifespan. Building on our detailed blood studies with over 30,000 participants^5^, we merged numerous open-source and collaborator datasets and performed integrated analyses across different organs. We characterized the landscape of age-related DMPs, VMPs and Shannon entropy dynamics across tissues and applied co-methylation network analyses and in silico perturbation simulations to identify key genes and pathways associated with epigenetic aging. Summary data and results are openly available at https://bioinformatics.erc.monash.edu/apps/human-aging-atlas/.

## Results

### A pan-tissue landscape of age-associated DNA methylation

We assembled a cross-sectional atlas of DNAm across 17 human tissues, totaling over 15,000 samples from 131 datasets (Supplementary Table 1). Global mean methylation ranged from 38% (cervix) to 63% (retina), though regional distributions were more uniform (Supplementary Table 2). Age-associated changes were evaluated using three complementary metrics: DMPs, VMPs and methylation Shannon entropy, each applied within individual datasets and meta-analyzed within tissues (Supplementary Data Fig. 1). DMPs were identified using multivariate linear regression, controlling for biological and technical covariates (for example, sex, body mass index (BMI) and batch). By contrast, VMPs were identified using a two-step Breusch–Pagan heteroscedasticity framework, detecting age-associated changes in methylation variance. Shannon entropy was computed from covariate-adjusted *β*values genome-wide and across CpG subsets and modeled as a function of age to capture sample-level epigenetic disorder. Together, these three layers provide complementary views of methylome remodeling, mean-level shifts, variance-level shifts and system-wide disorder across both locus-specific and tissue-wide scales.

### Multivariate linear analysis of age reflects distinct tissue-specific aging signatures

**Fig. 1:**
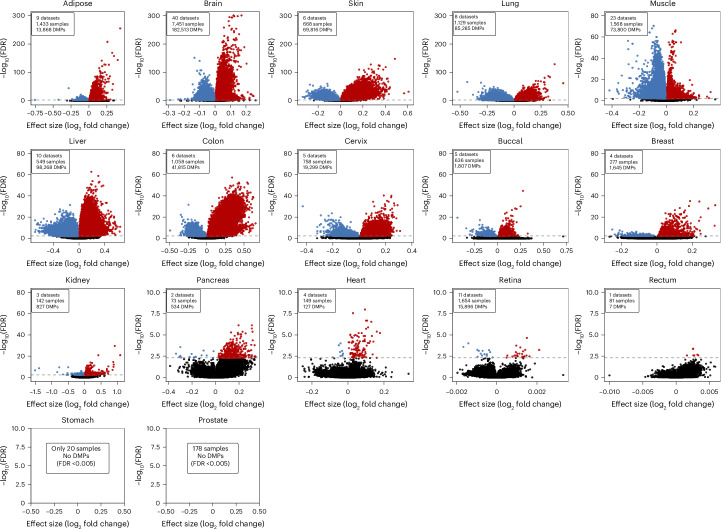
DMPs with age across 17 human tissues. Each volcano plot depicts a unique tissue and its methylation changes with age. Each dot corresponds to a distinct CpG; colored dots indicate a significant association with age at an FDR <0.005, while black dots indicate CpGs that do not exhibit significant age-associated changes.

Multivariate linear regression models, adjusting for relevant covariates, identified CpG sites significantly associated with chronological age across tissues. DMP counts (that is, age-associated CpGs) varied markedly (Fig. 1); brain, liver and lung exhibited the largest numbers (brain >180,000 DMPs), while pancreas, retina and prostate showed minimal or no significant age associations at a false discovery rate (FDR) <0.005. Some tissues, such as skin and the colon, exhibited strong signatures despite moderate sample sizes. All tissues except skeletal muscle and lung showed a predominance of age-associated hypermethylation.

**Fig. 2:**
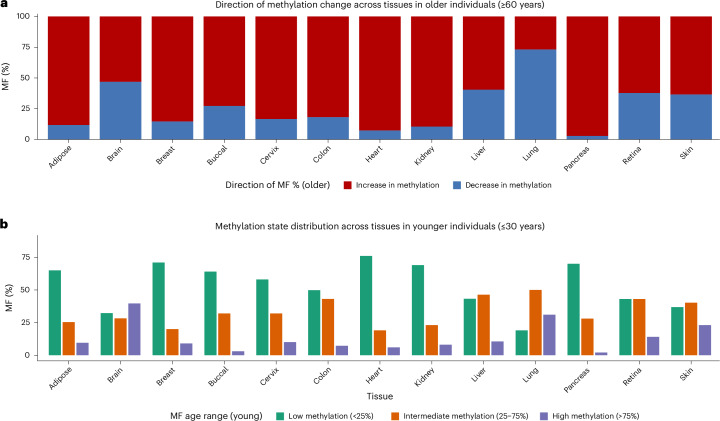
Methylation state and directional change of age-associated DMPs across 13 human tissues. **a**, The direction of methylation change in older individuals (>60 years). For each tissue, bars show the proportion of DMPs exhibiting increased methylation (red) or decreased methylation (blue) with age. **b**, The methylation-state distribution in younger individuals (<30 years) for the same DMPs. Bars show the proportion of DMPs classified as low methylation (<25%; green), intermediate methylation (25–75%; orange) or high methylation (>75%; purple). Tissues shown are adipose, brain, breast, buccal, cervix, colon, heart, kidney, liver, lung, pancreas, retina and skin. Prostate, rectum and stomach had insufficient samples from individuals under 30 years and are not included. For example, in adipose tissue, 13,868 age-associated DMPs were identified; in younger individuals for **b**, 65% showed low methylation, 25.3% showed intermediate methylation and 9.5% showed high methylation, while in older individuals for **a**, the majority of these DMPs showed increased methylation with age.

**Extended Data Fig. 1:**
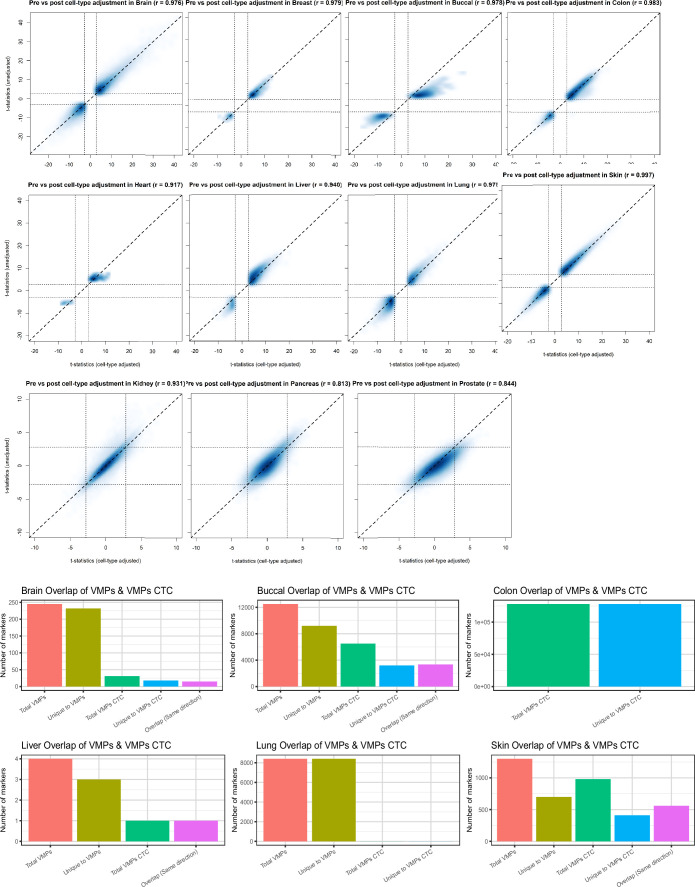
Effect of cell-type correction on age-associated methylation signatures across tissues. Correlation plots compare t-statistics for differentially methylated positions (DMPs) identified with and without cell-type correction across all tissues for which reference deconvolution panels were available. The x-axis shows t-statistics from cell-type corrected models and the y-axis shows t-statistics from uncorrected models. Pearson correlation coefficients (r) are shown for each tissue, with values ranging from r = 0.81 to r = 0.99, indicating that the majority of age-associated methylation signals are robust to cell-type composition correction. Bar plots show the number of variably methylated positions (VMPs) identified before and after cell-type correction per tissue. Cell-type proportions were estimated using EPISCORE for all tissues except buccal, where EpiDISH was used.

**Extended Data Fig. 2:**
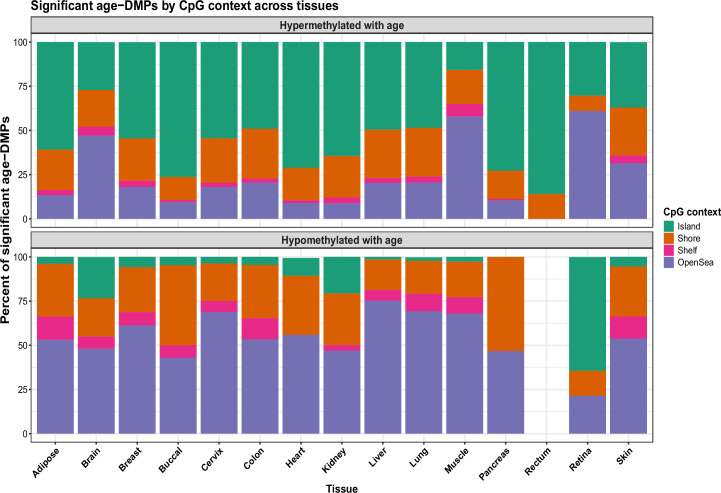
Distribution of age-associated DMPs across CpG island contexts. Significant age-associated CpGs (FDR < 0.005) were classified according to their genomic CpG context (Island, Shore, Shelf, and Open Sea) and stratified by direction of age association based on the sign of the effect size. Hypermethylated CpGs (increasing methylation with age) and hypomethylated CpGs (decreasing methylation with age) are shown separately. Bars represent the percentage of significant DMPs within each CpG context per tissue. Hypermethylated DMPs were more frequently located within CpG islands and shores, while hypomethylated DMPs were predominantly enriched in CpG-poor regions including shores and open sea across tissues.

The distribution of methylation fractions at DMPs between younger (<30 years) and older (>60 years) individuals revealed that CpGs with low (<25%) or intermediate (25–75%) methylation in youth predominantly exhibited age-associated hypermethylation, with some highly methylated regions (>75%) gaining further methylation with age (Fig. 2). Hypermethylated CpGs were more frequently located within CpG islands and shores, while hypomethylated CpGs were predominantly enriched in CpG-poor regions such as shores and open sea across tissues (Extended Data Fig. 1). Cell-type adjustment had a minimal impact on DMP detection, with *t*-statistics highly correlated between adjusted and unadjusted models (*r* = 0.81–0.99 across tissues), confirming that identified methylation signals are robust to cell-type composition (Extended Data Fig. 2 and Supplementary Table 3). Brain tissue was biologically distinct, showing a balanced distribution of hyper- and hypomethylated DMPs across all methylation fractions (28–40%), with neither direction predominating. The prostate, rectum and stomach lacked sufficient samples from individuals under 30 years to be included in this analysis.

**Extended Data Fig. 3:**
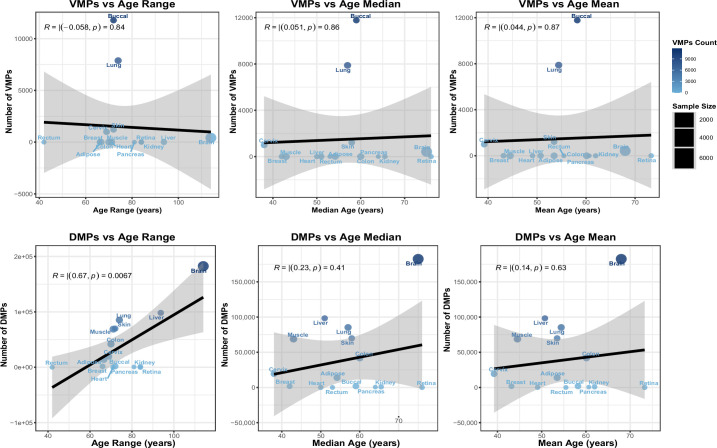
Sensitivity analyses examining the relationship between DMP and VMP detection and cohort characteristics across tissues. Top scatterplots showing the relationship between VMP count and three cohort characteristics: age range (R = −0.058, p = 0.84), median age (R = 0.051, p = 0.86), and mean age (R = 0.044, p = 0.87). No significant associations were identified, supporting the biological tissue specificity of VMPs rather than technical artifact. Bottom scatterplots showing the relationship between DMP count and the same cohort characteristics: age range (R = 0.66, p = 0.0072), median age (R = 0.22, p = 0.43), and mean age (R = 0.13, p = 0.65). Each point represents one tissue dataset. Only age range showed a significant association with DMP yield, indicating that broader lifespan sampling enhances statistical power to detect age-related methylation changes without introducing systematic bias related to mean or median age.

Sensitivity analysis confirmed that DMP yield correlated significantly with age range (*R* = 0.66, *P* = 0.0072) but not mean or median age (*R* = 0.13 and 0.22, respectively), indicating that broader lifespan sampling enhances detection power without introducing systematic bias (Extended Data Fig. 3b). Power analysis^15^ revealed that limited DMP discovery in tissues such as heart, pancreas, prostate and stomach reflects insufficient sample sizes rather than biological stability, as detectability for median effect sizes was extremely low at FDR ≤0.005 in these tissues (Supplementary Table 4). By contrast, brain tissue (*N* = 7,451) demonstrated high power for upper-quartile effects, resulting in substantially more significant associations. Lung and liver tissues showed comparatively higher detectability (61% and 49%, respectively) despite moderate sample sizes, reflecting comparatively larger effect sizes and favorable variance structure. Skeletal muscle (*N* = 1,568), despite an adequate sample size, exhibited smaller effect sizes overall, leading to more modest detectability. Together, tissue-specific discovery rates are driven by both sample size and the underlying distribution of effect sizes.

### VMPs are scarce and highly tissue-specific

**Fig. 3:**
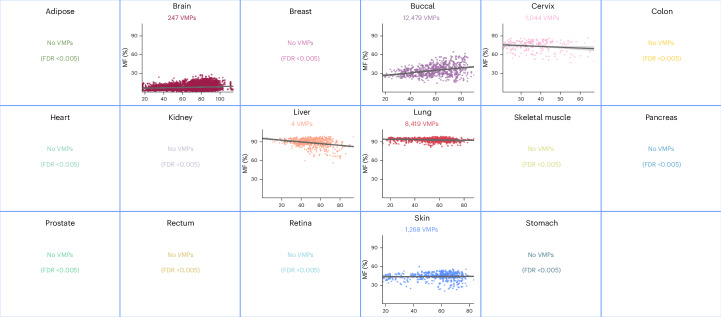
Age-related changes in VMPs across 17 human tissues. Each scatter plot illustrates age-associated VMPs for a specific tissue. Only brain, buccal, cervix, liver, lung and skin tissues had significant age-associated VMPs at FDR <0.005; the remaining 11 tissues showed no significant VMPs and are displayed for reference. Each dot represents a unique individual, with the *x* axis showing age and the *y* axis showing methylation level (*β* value). Individual data points are shown throughout; no summary statistics or error bars are presented.

**Extended Data Fig. 4:**
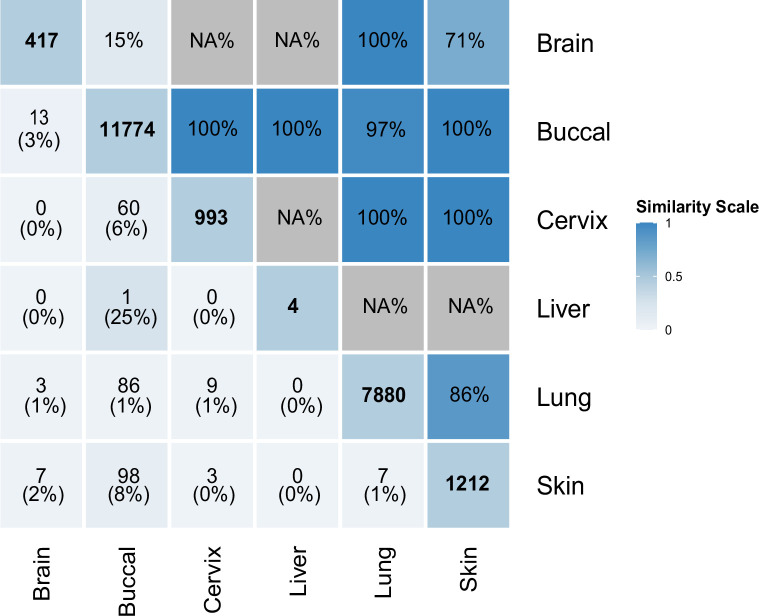
Cross-tissue overlap of age-associated VMPs reveals predominantly tissue-specific aging signatures. Combined similarity heat map of pairwise VMP overlap across 17 human tissues. The lower diagonal shows pairwise Jaccard indices of VMP overlap; darker blue indicates greater overlap, with hierarchical clustering revealing groups of tissues with shared age-associated VMPs. Most tissues show low overlap, reflecting predominantly tissue-specific VMP patterns consistent with locally stochastic epigenetic drift. The upper diagonal shows effect-size correlation across tissues to assess the directionality concordance of overlapping VMPs; darker red indicates greater concordance in the direction of variance change. Non-chance overlaps were identified between brain–buccal, buccal–cervix, buccal–lung, brain–skin, and buccal–skin tissue pairs based on permutation testing (Supplementary Table 5).

Compared with DMPs, VMPs were much less frequent across tissues. Only brain, buccal, cervix, lung and skin had substantial numbers of age-VMPs, whereas most tissues showed few or none at FDR <0.005 (Fig. 3). Cross-tissue VMP overlap was limited, with fewer than 100 VMPs shared between any tissue pair (Extended Data Fig. 4). To determine whether the observed overlap of age-associated VMPs across tissues exceeded what would be expected by chance, we performed a permutation-based analysis. For each tissue comparison, we randomly selected sets of CpGs matched in number and genomic distribution to the observed VMPs and repeated this process 1,000 times to generate a null distribution of overlaps. The actual observed overlaps were then compared with this distribution to compute empirical *P* values. This analysis revealed that half of the overlaps between tissue pairs occurred by chance, except for the pairs involving brain and buccal, buccal and cervix, buccal and lung, brain and skin, and buccal and skin (Supplementary Table 5a). VMP detection showed no significant correlation with age range, mean age, median age or sample size (*R* ≈ 0.05, *P* > 0.8), supporting biological tissue specificity rather than technical artifacts (Extended Data Fig. 3a).

Among nonchance overlaps, most shared VMPs showed concordant directionality and similar trends, such as increases or decreases in variability (Extended Data Fig. 4). An exception was brain–buccal VMPs, where only 15% of the 13 overlapping VMPs were directionally consistent. Pathway enrichment analysis showed no enriched pathways for most individual tissues, except for buccal and lung tissues. Buccal VMPs were enriched across 210 pathways (FDR <0.005), particularly in system development (Supplementary Table 6), while in lung tissues, VMPs were enriched in only three pathways: external encapsulating structures, the extracellular matrix and collagen-containing extracellular matrices. VMPs were particularly sensitive to cell-type correction in lung and liver, while colon displayed increased VMPs following adjustment (Supplementary Table 3 and Supplementary Fig. 1). Collectively, these results demonstrate that age-associated hypermethylation is a consistent feature across tissues, with the contribution of cellular composition varying by tissue.

### Shannon entropy and epigenetic disorder reveal tissue-specific aging patterns

**Fig. 4:**
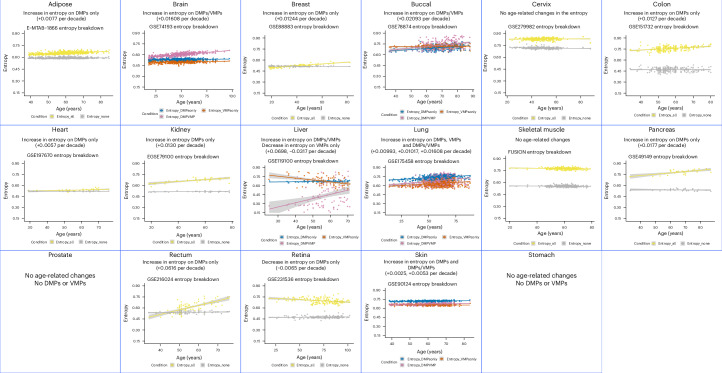
Shannon entropy of DNAm with age across 17 human tissues. Each plot represents a specific tissue using the largest available datasets. Tissues are grouped into three categories on the basis of the presence of age-associated DMPs and VMPs. Tissues without VMPs (adipose, breast, cervix, colon, heart, kidney, skeletal muscle, pancreas, rectum and retina) show two conditions: ‘Entropy_all’ (entropy calculated across all CpGs genome-wide; shown in yellow) and ‘Entropy_none’ (entropy calculated across CpGs not associated with age; shown in gray). Tissues with both DMPs and VMPs (brain, buccal, liver, lung and skin) show three conditions: ‘Entropy_DMPsonly’ (entropy at CpGs that are DMPs only; shown in blue), ‘Entropy_VMPsonly’ (entropy at CpGs that are VMPs only; shown in orange) and ‘Entropy_DMPVMP’ (entropy at CpGs that are both DMPs and VMPs; shown in pink). Prostate and stomach tissues showed no age-related DMPs or VMPs and are therefore not represented. Lines show fit regression of entropy against age; shaded bands represent 95% confidence intervals.

Shannon entropy captures the overall disorder and unpredictability of DNAm patterns across the genome, offering a single quantitative measure that integrates all age-related differential changes including those from low-variability DMPs. While VMPs shift entropy toward higher or lower states, many of these CpGs are already intermediately methylated^16^ in youth, meaning entropy dynamics are not strictly age-dependent at these sites. This analysis therefore provides a complementary layer of insight beyond DMP- or VMP-based approaches, revealing tissue-specific trajectories of epigenetic disorganization during aging (Fig. 4).

Entropy increased preferentially at DMPs in several tissues, such as adipose, breast and kidney, suggesting the progressive loss of epigenetic fidelity at aging-associated sites, consistent with models of epigenetic drift in metabolically active and proliferative tissues^4,5,17^. By contrast, brain, buccal epithelium, liver, lung and skin tissues exhibited entropy increases at positions that were both differentially and variably methylated (that is, DMP–VMP overlaps), indicating a more stochastic mode of epigenetic aging in these tissues, where both directionality and variability of methylation are disrupted.

### WGCNA highlights tissue-specific hubs and conserved aging modules

**Extended Data Fig. 5:**
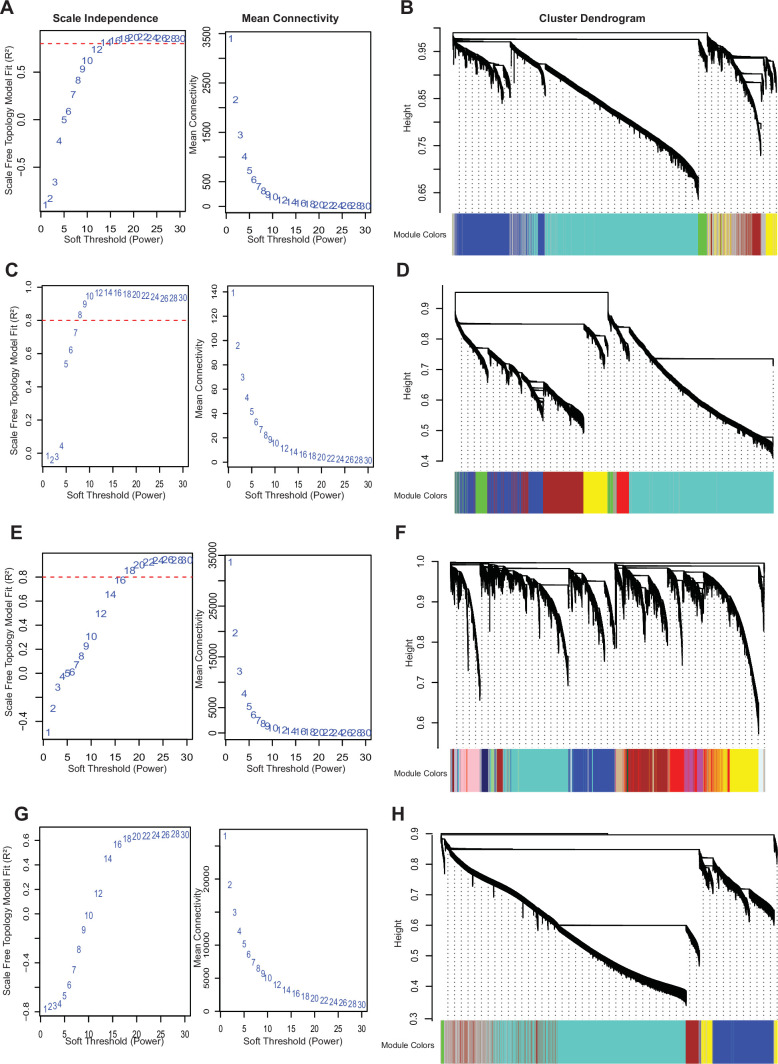
WGCNA soft-thresholding power selection and co-methylation modules identification across tissues. (**A**,**C**,**E**,**G**) Scale-free topology fit index (y-axis) plotted against a range of soft-thresholding powers (x-axis) for skeletal muscle, adipose tissue, blood, and brain respectively. The selected soft-thresholding power corresponds to the value at which the scale-free topology fit index approached or exceeded R^2^ ≥ 0.85, indicating a robust scale-free network structure. Mean connectivity across the same range of powers is also shown, illustrating the trade-off between network sparsity and connectivity used to guide power selection. (**B**, **D**, **F**, **H**) Hierarchical clustering dendrograms of CpGs grouped by topological overlap matrix for each tissue, with module colours representing distinct co-methylation modules identified by dynamic tree cutting. Each branch of the dendrogram corresponds to a module of highly co-methylated CpGs. The number and size of modules varied across tissues, reflecting tissue-specific biological complexity.

**Extended Data Fig. 6:**
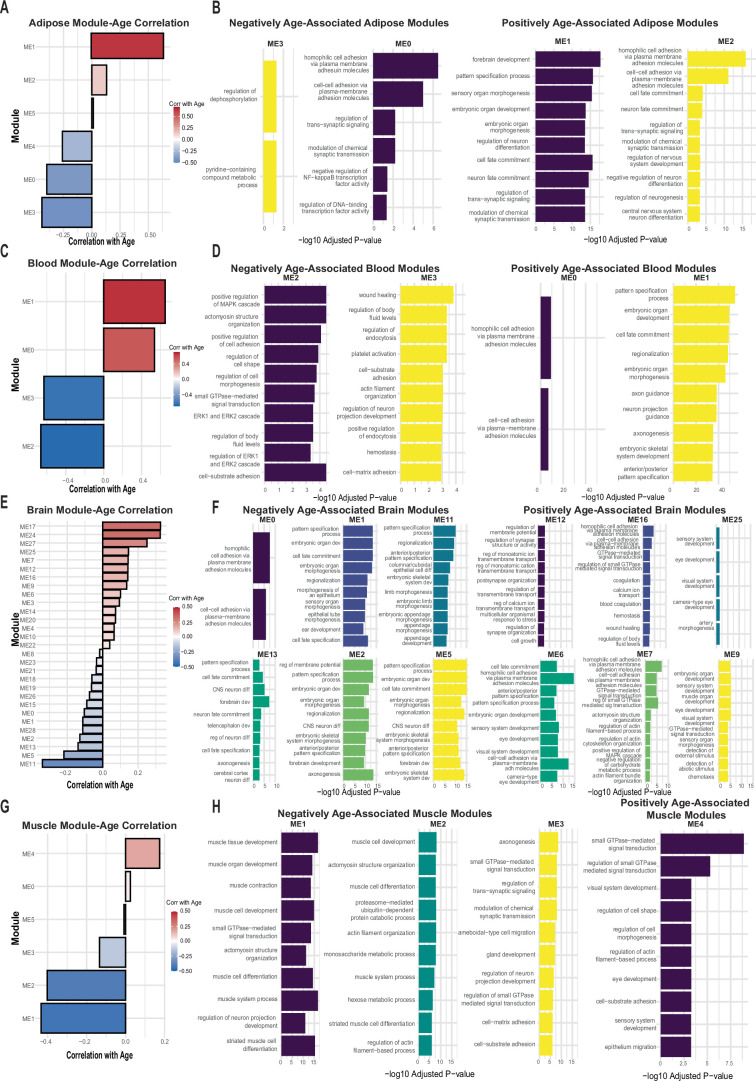
WGCNA module eigengene correlations with age and pathway enrichment across four tissues. (**A, C, E, G**) Module eigengene–age correlation plots for skeletal muscle, adipose tissue, blood, and brain respectively. Each row represents a co-methylation module, and each column represents age correlation statistics. Modules are classified as positive (hypermethylated with age, shown in red) or negative (hypomethylated with age, shown in blue) based on the direction of the module eigengene–age correlation. Only statistically significant modules (p < 0.05) were retained for functional interpretation. (**B, D, F, H**) Dot plots showing the top enriched Gene Ontology (GO) biological process pathways for positive and negative age-associated modules in each tissue. Dot size reflects the number of genes associated with each pathway and dot colour indicates FDR strength; more saturated colours indicate stronger enrichment. Pathways are separated by module direction to facilitate comparison of hypermethylated and hypomethylated aging programs within each tissue.

To characterize co-regulatory methylation frameworks across tissues, we applied weighted gene co-expression network analysis (WGCNA) to skeletal muscle, adipose tissue, blood and brain methylation data, constructing tissue-specific co-methylation networks. Each tissue generated specific modules, with eigengene expression trajectories showing distinct aging signatures (Extended Data Fig. 5a,c,e,g). Subsequently, we constructed a signed network and conducted hierarchical clustering on the topological overlap matrix to identify modules. The number and size of modules varied among tissues, reflecting biological complexity in a data-driven approach (Extended Data Fig. 5b,d,f,h). Module eigengenes, the first principal component of each module, were calculated and correlated with age to identify those with strong associations with aging. Significant positive and negative age-associated modules were included for further functional interpretation (Extended Data Fig. 6a,c,e,g).

**Fig. 5:**
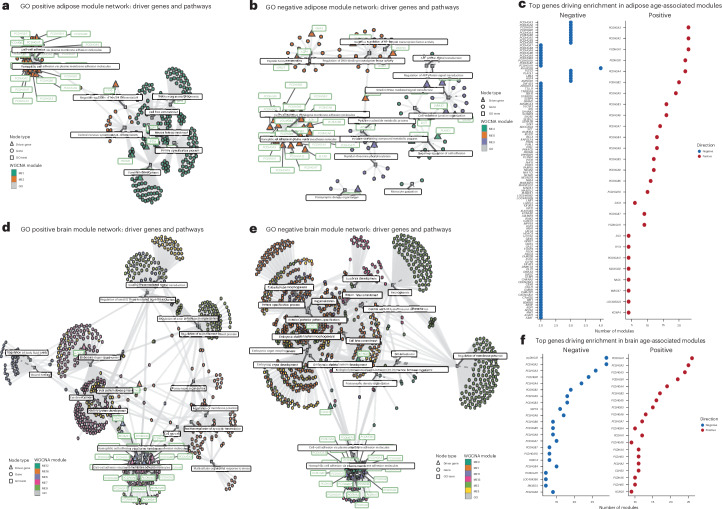
WGCNA co-methylation network analysis across four tissues. **a**,**b**,**d**,**e**, The leading enriched pathways for positive (hypermethylated with age; **a**,**d**) and negative (hypomethylated with age; **b**,**e**) co-methylation modules in adipose tissue (**a**,**b**) and brain (**d**,**e**), respectively. Each panel corresponds to a distinct tissue, with colors indicating module affiliation. Those identified as module-influential are highlighted in green and represented as triangles; associated pathways are highlighted in gray. **c**,**f**, Dot plots of the frequency with which individual genes were ranked among the top module-influential genes across age-associated WGCNA modules for adipose tissue (**c**) and brain tissue (**f**), stratified by direction of module–age associations. The *y* axis indicates gene names and the *x* axis reflects the number of pathways influenced by each gene within its module. kME was used to define module-influential genes as those with the highest intramodular connectivity and strongest association with age. Recurrently identified module-influential genes represent network-central loci consistently implicated in aging-associated methylation programs across tissues.

Gene Ontology (GO) biological processes categorized the enriched pathways according to module direction, allowing us to distinguish between age-related increases in methylation (positive modules) and decreases in methylation (negative modules). In adipose tissue, age-associated positive modules exhibited heightened methylation at loci related to forebrain development and cell adhesion, while hypomethylated modules were enriched for synaptic signaling and NF-κB regulation, indicating a potential loss of regulatory control in immune and neuronal pathways (Extended Data Fig. 6b). In blood, hypermethylated modules were related to embryonic development, cell-adhesion, apoptotic signaling and immune pathways, while hypomethylation modules reflected homeostasis regulation, including cell shape and regulation of body fluid levels (Extended Data Fig. 6d). The brain displayed increased methylation in modules enriched for GTPase activity, synaptic activity and organization, cell communication and adhesion, among others, while decreased methylation impacted multiple morphogenesis-related processes and developmental pathways (Extended Data Fig. 6f). In skeletal muscle, gains in methylation were observed in GTPase signaling pathways and some nonmuscle developmental pathways. At the same time, losses were noted in pathways regulating muscle development and differentiation, as well as metabolic-related pathways (Extended Data Fig. 6h). Beyond tissue-specific patterns, we also identified converging modules that revealed shared epigenetic aging programs across tissues. Across tissues, homophilic cell adhesion via plasma membrane adhesion molecules was enriched in adipose, blood and brain tissue (Fig. 5a,b,d,e), implicating a common loss of cell–cell interaction integrity during aging. Small GTPase-mediated signal transduction was enriched in both brain and skeletal muscle, indicating conserved age-associated changes in intracellular communication and cytoskeletal regulation.

Module membership (kME), defined as the correlation between a gene’s methylation profile and the module eigengene, was used to identify module-influential genes, those exhibiting both high intramodular connectivity and strong association with age. Across all tissues (that is, adipose and brain), members of the protocadherin gamma (PCDHG) gene family were consistently identified as primary module-influential genes (Fig. 5c,f). While PCDHG genes are traditionally associated with neural development and synaptic patterning, their consistent epigenetic regulation in nonneural tissues, including blood and skeletal muscle, suggests a broader role in maintaining structural and signaling stability across organ systems during aging.

### Cross-tissue overlap reveals core and divergent epigenetic aging programs

**Fig. 6:**
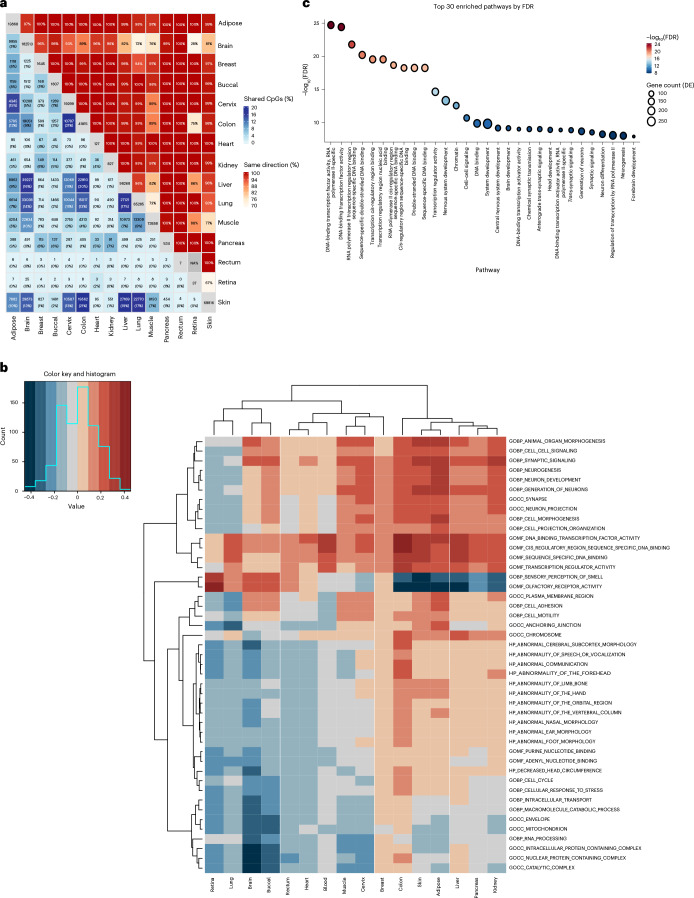
Cross-tissue overlap of age-associated DMPs reveals both conserved and tissue-specific aging signatures. **a**, A combined similarity heat map of pairwise DMP overlap across 17 human tissues. The lower diagonal shows pairwise Jaccard indices of DMP overlap; darker blue indicates greater overlap. The upper diagonal shows effect-size correlation across tissues to assess directionality concordance of overlapping DMPs; darker red indicates greater concordance. **b**, Pathway enrichment analysis of CpGs identified as DMPs in nine or more tissues. Dot color indicates FDR strength and dot size reflects the number of genes associated with each pathway. **c**, A heat map of the 50 most enriched pathways from the mitch multi-tissue pathway enrichment analysis. Red indicates hypermethylation and blue indicates hypomethylation of genes within each pathway. Enriched pathways are listed on the *y* axis and tissues are shown on the *x* axis.

To determine whether epigenetic aging signatures are consistent across tissues or predominantly tissue-specific, we analyzed the overlap of DMPs across all tissues in our atlas. We also included the blood DMPs identified in our previous blood atlas publication^5^. The intersection heat map (Fig. 6a, bottom diagonal half) depicts a complex scenario, showing that DMP overlap between tissues varies from 0% to 25% of the total DMPs, as determined by the tissue with the fewest DMPs. This suggests that most pairwise comparisons show a restricted number of common DMPs, emphasizing distinct aging pathways unique to each tissue, or possibly reflecting a reduced ability to detect DMPs, especially in tissues with limited samples. When evaluating the directionality of the intersected DMPs across tissues (Fig. 6a, top diagonal half), a majority exhibit changes in the same direction, with direction concordance exceeding 59% for all tissue pairs except the brain and retina. The brain and retina demonstrated the highest level of discordant directionality, with only 28% of the 25 overlapping DMPs moving in the same direction. In our search for DMPs with the greatest overlaps among tissues, we identified cg16867657 as significant in 15 tissues (excluding the rectum). This specific CpG site is located on chromosome 6 at position 11044643–11044645, harboring the well-known aging-related gene *ELOVL2*. A total of 37 DMPs were identified across 13 or more tissues (Supplementary Table 9).

To ensure that the observed overlaps were not merely due to random chance, we performed permutation tests (as described in the Methods), by randomly rearranging the sample age labels within each tissue and recalculating DMP overlaps over 1,000 iterations. As shown in Supplementary Table 5b, the results indicated that the empirical overlaps between several pairs of tissues significantly surpassed the 95th percentile of the null distribution, providing compelling evidence against coincidental concordance. The exceptions were buccal versus rectum, cervix versus retina, liver versus retina, rectum versus retina, retina versus blood, and retina versus skin. Notably, only one dataset was found in the analysis of the retina and rectum, which significantly limits the ability to identify age-related changes in these tissues, probably resulting in observed overlaps that do not meet the null distribution.

Finally, we conducted a sensitivity analysis to confirm that our DMPs’ identification was not influenced by technical artifacts such as age ranges, mean age or median age. The updated scatter plots examining DMPs versus age (Extended data Fig. 3b) illustrate our tests for correlations between the number of DMPs and the age range, median age and mean age across different tissues. Our analysis revealed that only the age range demonstrated a significant correlation with DMP count (*R* = 0.66, *P* = 0.0072), while mean and median age showed no significant correlation (*R* = 0.13 and 0.22, respectively). This suggests that a broader sampling across the lifespan enhances detection power without introducing systematic bias.

### Cross-tissue network level analysis through module enrichment identifies key pathways and genes as module-influential genes in aging

We subsequently investigated the functional significance of genes that consistently showed age-related changes in methylation across various tissues. We established a rigorous consensus set of genes differentially methylated in nine or more tissues (>50% overlap across tissues) and conducted pathway enrichment analysis on this core group. Notably, a prominent signature surfaced: almost all enriched pathways were associated with transcriptional regulation, chromatin organization and gene regulation (Fig. 6b).

While compelling, this method has constraints owing to statistical power; tissues with fewer samples or minor methylation changes may not be adequately represented (Supplementary Table 1). In addition, it restricts interpretability, as it does not enable comparison of directionality across tissues in each pathway. To tackle these issues, we employed mitch^18^, which combines the magnitude and direction of gene-level shifts across different tissues using *t*-statistics rather than depending only on significance thresholds. This approach allowed underpowered tissues to meaningfully contribute to the overall analysis landscape. The integration based on mitch essentially validated the transcriptional regulation signature while broadening it to include additional pathways relevant to aging, such as mitochondrial function, cell-cycle pathways and DNA damage response (Supplementary Table 7). A thorough examination of the top 50 pathways has unveiled three distinct clusters that illustrate a consistent shift in agreement across nearly all tissues (Fig. 6c). For instance, pathways associated with transcription factor activity, as observed in the analysis above, DNA binding and transcriptional regulation were identified as being enriched in hyper-DMPs across all tissues. In addition, pathways related to cell–cell signaling, synaptic signaling and neurogenesis were observed to be enriched in hyper-DMPs across all tissues, with the exception of the retina and lung. Conversely, pathways involved in intracellular processes, such as those pertaining to mitochondria and macromolecule metabolism, were found to be enriched in hypo-DMPs in all tissues, except for breast and colon.

To further investigate the signatures identified by mitch, we generated a gene–gene correlation matrix across tissues utilizing Spearman correlation. This method evaluated the consistency of each gene’s aging profile across various tissues. For instance, if two genes increase in methylation levels with age in most tissues, they are considered to have a strong positive correlation. Conversely, if the methylation of one gene increases in some tissues while in another decreases, the correlation is regarded as negative. Consequently, we were able to model ‘*t*-patterns’, indicating whether a gene is uniformly upregulated or downregulated with age across tissues, or exhibits fluctuations that are specific to certain tissues. We utilized hierarchical clustering and dynamic tree cutting on the gene–gene correlation matrix to extract biological structures from these patterns, grouping genes into modules on the basis of co-aging behavior. Unlike WGCNA modules, these modules arise from cross-tissue coherence in aging trajectories as indicated by *t*-statistics, rather than solely from co-methylation patterns in a single tissue.

**Extended Data Fig. 7:**
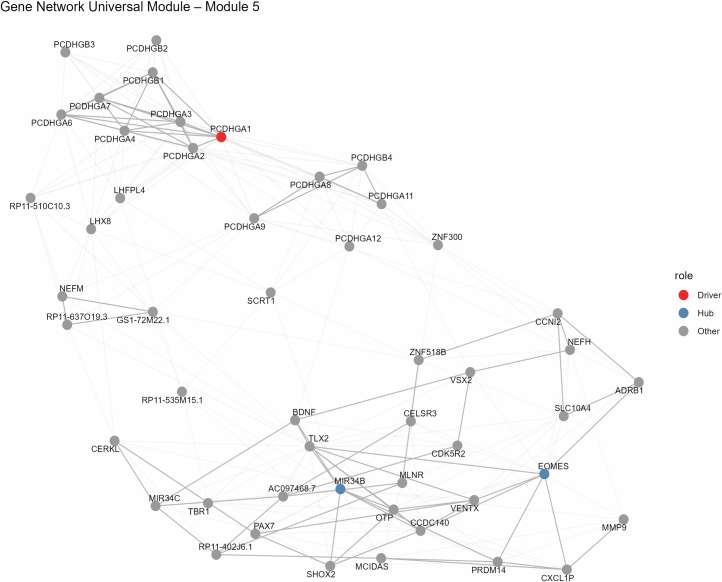
Cross-tissue perturbation analysis network of universal co-methylation module identified across all tissues (Module 5). Each node represents a gene within the module, with edges reflecting the strength of gene–gene correlation across tissues; edge thickness is proportional to correlation strength. Module-influential genes, defined by high intramodular connectivity (kME) combined with strong average t-statistic signal across tissues, reflecting consistent age-associated methylation changes, are shown in red and represent genes most likely to have functional impact on aging-associated methylation programs. Hub genes, defined by high correlation strength to other genes within the module, functioning as coordinators or integrators of module-level co-methylation activity, are shown in blue. *PCDHGA1*, a member of the Protocadherin Gamma (*PCDHG*) gene family, was identified as the top module-influential gene in this universal module, consistent with its cross-tissue role in aging-associated methylation networks.

Each resulting module captures a group of genes that age uniformly across different tissues. Some modules exhibited significantly high positive *t*-scores in nearly all tissues, indicating a widespread hypermethylation linked to aging (Supplementary Table 6). By contrast, other modules were distinctly tissue-specific, showing aging-related hypermethylation in one tissue while demonstrating hypomethylation in another, suggesting varying aging mechanisms. This analysis uncovered several important findings: for instance, module 2 showed elevated average *t*-statistics in blood, indicating significant hypermethylation associated with aging, while also revealing negative *t*-statistics in the brain, suggesting hypomethylation. Only one module (module 5) exhibited a universal pattern, indicating hypermethylation across all tissues (Extended Data Fig. 7 and Supplementary Table 6). Importantly, the module-influential gene in this module belongs to the *PCDHG* gene family (*PCDHGA1*), reinforcing the idea that this gene family may play a key role in aging processes throughout all human tissues. In summary, our findings highlight that specific gene programs (module clusters) are regulated in a highly tissue-specific manner during aging, while others may indicate shared core aging mechanisms. This may also present tissue-specific or even contradictory regulations (for example, beneficial in one tissue but harmful in another).

### In silico robustness analysis reveals functionally fragile genes driving module architecture

To advance from correlation to testing functional effects, we established an in silico validation framework. In this framework, we computationally adjusted methylation levels for each gene, simulating conditions, such as hypermethylation, and reassessed the structural integrity of aging-associated modules. This simulation-driven strategy identified a limited set of genes (defined here strictly in terms of network topology and simulated mathematical disruption, not biologically validated causal effects) that modified the arrangement of various modules across different tissues (Supplementary Table 7). The *PCDHG* gene family, *MEST*, *HDAC4* and genes from the *HOX* family were predominant in the set of genes identified. When combined with additional key genes from each module, they instigated significant changes in modular connectivity, with some modules exhibiting alterations exceeding 50% when key genes were modified. Considering the enrichment pathways of each module, it is reasonable to assert that alterations in DNAm result in changes in function pertinent to these pathways.

To better understand the biological effects of these modulations, we integrated module-level enrichment findings and classified gene modifications as potentially beneficial or detrimental. For instance, if a gene affects a module linked to aging pathways and the effect diminishes the module’s influence, it is deemed beneficial; conversely, if the modulation enhances the module’s effect, it is considered harmful. Only ten modules presented a beneficial effect, while all others appear to have a harmful effect if modified. These findings suggest that most aging-related modules are vulnerable to modifications, with only a small subset showing potential for beneficial modulation.

## Discussion

This meta-analysis of over 15,000 methylomes across 17 human tissues, demonstrates that aging is associated with both tissue-specific and conserved cross-tissue effects on the methylome, characterized across three complementary layers: DMPs, VMPs and Shannon entropy. The heterogeneity of DMPs across tissues reflects the organ-specific nature of the aging process^19–21^.

Tissues such as the brain, liver, lung, skeletal muscle and skin exhibited a large number of DMPs associated with age, suggesting a reconfiguration of the methylome or vulnerability associated with advanced age^4,22,23^. By contrast, tissues such as the kidney, prostate, rectum and stomach demonstrated minimal to no detectable changes, probably attributed to the limited sample size or an inherently more stable epigenetic landscape. However, power analyses indicated extremely low detectability for median effect sizes in these tissues at FDR ≤0.005, largely owing to small sample sizes and the resulting stringent empirical significance thresholds. These findings suggest that the limited number of DMPs observed in these tissues more likely reflects insufficient statistical power rather than a biologically stable epigenetic landscape. Larger cohorts will be required to determine whether comparable age-associated methylation changes are present in these rarer tissue types. Despite this tissue variability, a discernible pattern emerged: most tissues demonstrated age-associated hypermethylation, particularly in previously unmethylated regions in younger individuals^22–24^. This observation implies a transition toward epigenetic silencing, probably resulting from the closure of open chromatin regions, affecting enhancer activity or gene expression regulation^24,25^. Exceptions to this trend, were skeletal muscle and lung tissues, which demonstrated heightened hypomethylation, which may signify tissue-specific aging mechanisms^6,26,27^, encompassing demethylation at regulatory or structural loci.

The predominance of age-associated hypermethylation across multiple tissues should not be interpreted (on its own) as evidence against stochastic epigenetic drift. Because many tissues in the younger cohort showed a predominance of low methylation states, age-associated increases in methylation are, in many cases, compatible with drift toward more intermediate methylation levels (Supplementary Table 8). To address this, we examined methylation-state transitions between young and older groups in parallel with the direction of methylation change. This comparison suggests that, in several tissues, a substantial fraction of age-associated increases probably reflects movement toward intermediate methylation states, consistent with entropic drift, whereas in others, the patterns may also be compatible with more structured regional remodeling. In addition, although some age-associated signal was attenuated after deconvolution, many DMPs persisted, indicating that both cell-compositional changes and cell-intrinsic methylation alterations contribute to tissue aging. Together, these findings support a model in which age-related methylation change reflects a mixture of stochastic drift and tissue-specific epigenetic remodeling, rather than a single uniform process^7,20,21,28^. This insight expands upon previous aging epigenome studies that primarily quantified methylation changes as a binary gain or loss, and highlights the importance of baseline methylation state in shaping the trajectory of age-associated epigenetic remodeling. Future studies should investigate whether these directional shifts correspond with chromatin accessibility and histone modifications, and whether they confer functional consequences on regulatory landscapes relevant to tissue aging and disease vulnerability.

Unlike DMPs, VMPs are rare and highly tissue-specific, supporting the idea that an aging-related epigenetic drift occurs randomly and locally^4,5,29,30^. Few tissues show many VMPs, and most lack consistent pathway enrichment, except buccal and lung tissues, implying these changes are probably random degradation rather than biological responses^7,31^. This questions the view that methylation variability is always harmful or meaningful in aging, emphasizing the need to distinguish noise from genuine signals^29^. Entropy, linked to molecular chaos, varies by tissue and is highest in metabolically active organs such as adipose, breast and kidney, possibly indicating regulatory loss in energy-demanding tissues^7,32,33^. The brain’s entropy profile is unique, balancing DMPs and VMPs, hinting at a different aging process that may reduce both directional and random methylation changes^7,17^.

It is well established that a subset of CpGs is under genetic control^34^ methylation quantitative trait locus and may exhibit higher inter-individual variability. However, our VMP analysis tested for age-associated changes in variance rather than baseline variability per se. Because methylation quantitative trait locus effects are generally stable across the lifespan, they are unlikely to account for systematic age-dependent variance shifts unless interacting with age. Thus, while genetic regulation may influence absolute methylation variability, the VMPs identified here reflect loci where variance changes with age.

Our WGCNA analysis identified modules of co-methylated CpGs with distinct aging patterns and functions. Modules with higher methylation in older individuals were linked to developmental and adhesion pathways, while those with decreased methylation related to immune, metabolic and neurogenic signaling. This reflects aging hallmarks: reduced regenerative capacity and altered cell communication. GTPase signaling and cell-adhesion pathways were enriched across tissues, indicating aging affects synaptic and cytoskeletal functions. Small GTPases such as Rho, Ras and Rab are key in cytoskeletal, vesicle, and cell communication activities that decline with age. Dysregulation of GTPases is linked to neurodegeneration, immunosenescence and senescence^7,35–37^. Growing interest in drugs targeting GTPases as gerotherapeutics supports our findings, demonstrating their relevance.

The PCDHG gene family emerged repeatedly as a module-influential candidate across the tissue-specific WGCNA network and the pan-tissue analysis. PCDHG genes encode cell-adhesion proteins critical for synaptic organization^38^, and hypermethylation in this family has been associated with reduced brain white matter^39^, a marker of cognitive decline. This connection is intriguing as previous studies show abdominal fat can predict decreases in cerebral white matter^28,40^. This gene family is also associated with age-related diseases, including increased inflammation, reduced stroke volume, pre-cancerous lesions^41^, cancers and muscle weakness^42^. Identified as key candidates across tissues, modifications in the DNAm of these genes may impact aging pathways. Our in silico simulation shows that manipulating this gene family affects 100% of its module, influencing aging pathways. Overall, these genes are central in aging-related networks, and our findings suggest tissue-specific methylation patterns could serve as biomarkers and therapeutic targets.

Pan-tissue DMPs in *ELOVL2*, *KLF14*, *FHL2*, *TBR1* and *TRIM59* confirm systemic aging biomarker candidates, with permutation testing confirming these overlaps are unlikely due to chance. DMPs often showed conserved directionality across tissues, even with minimal overlap, suggesting a balance between global conservation and tissue specificity that may drive systemic aging features such as frailty^43^. Genes showing consistent cross-tissue directionality represent candidates for systemic biomarkers, while tissue-specific DMPs and modules may provide more precise markers of organ vulnerability relevant to neurodegeneration, metabolic dysfunction and muscle decline.

Our mitch-based meta-analysis expanded the picture by integrating gene-level statistics across tissues. This revealed broad upregulation of pathways tied to transcriptional regulation and chromatin remodeling (for example, DNA binding and histone modification), as well as age-related suppression of metabolic and mitochondrial processes. Notably, transcriptional repression via DNA hypermethylation emerged as a conserved hallmark, while intracellular metabolic decline via hypomethylation was evident in all tissues except breast and colon. This suggests an imbalance between nuclear and cytoplasmic aging programs, a theme increasingly recognized in aging biology^44^.

Perturbation modeling identified *PCDHGA1*, *MEST*, *HDAC4* and HOX family genes as having the highest cross-module perturbation scores, with most perturbations worsening aging-associated module profiles. This suggests that the majority of aging co-methylation networks are fragile and resistant to beneficial modulation. A notable exception was a resilient module enriched for NAD^+^ biosynthesis via the nicotinamide riboside salvage pathway. NAD^+^ declines with age, contributing to mitochondrial dysfunction, reduced sirtuin activity and accumulating DNA damage. Nicotinamide riboside supplementation has been shown to counteract age-related decline in tissues such as brain^45^, muscle^46^ and heart^47^, supporting the NAD^+^ salvage pathway as a therapeutically relevant node in the aging methylome.

### Conclusions and limitations

Our study provides a cross-tissue characterization of human epigenetic aging across three complementary methylation layers: DMPs, VMPs and Shannon entropy. The data support a model in which aging reflects both stochastic variation and structured, tissue-dependent methylome remodeling rather than uniform random drift. Cross-tissue and in silico analyses identify module-level fragility within aging-associated pathways and highlight resilient modules, particularly those enriched for NAD^+^ biosynthesis, with potential therapeutic relevance. Summary data and analytical results are openly available at https://bioinformatics.erc.monash.edu/apps/human-aging-atlas/.

Nevertheless, certain limitations should guide interpretation. Unequal sample sizes across tissues may limit detection power in underrepresented organs (Supplementary Table 1), and furthermore, the use of bulk tissue masks cell-type-specific signals and compositional shifts. Accordingly, interpretations of tissue-specific methylation levels, particularly those in the intermediate range, should be viewed with caution, as they may reflect differences in cellular composition rather than purely intrinsic epigenetic remodeling. The cross-sectional nature of the data constrains our ability to capture temporal trajectories of epigenetic aging. It therefore cannot directly distinguish within-individual aging effects from cohort-related differences. However, the limited availability of longitudinal epigenomic data across human tissues, particularly for solid tissues, represents a field-wide constraint. Our findings should thus be interpreted as cross-sectional aging-associated signatures, with longitudinal validation remaining an important future direction. Furthermore, the absence of complementary omics layers, such as chromatin accessibility or gene expression, limits mechanistic insight. Although computational predictions offer testable hypotheses, experimental validation is essential to confirm causal roles for identified genes and modules. Despite these challenges, our atlas sets the stage for deeper, integrative studies of the aging methylome, and offers a scalable framework for understanding and potentially modulating human aging at the epigenetic level.

## Methods

### Ethics statement

All original studies included in this meta-analysis were conducted in accordance with the Declaration of Helsinki. Each contributing dataset was collected under ethical approval from the relevant institutional review board or ethics committee at the originating institution, and written informed consent was obtained from all participants. For datasets collected by the authors’ institutions, ethical approval was granted by the Human Research Ethics Committee of Victoria University (Melbourne, Australia). As this study constitutes a secondary analysis of publicly available and collaborator-shared data, no additional ethical approval was required.

### Study design and scope

This study represents a large-scale, cross-tissue epigenome-wide association meta-analysis of human aging, integrating over 15,000 methylomes from 17 different tissues. Given the typically small effect sizes of age-related DNAm shifts, meta-analyses can be used to overcome these limitations and enhance reproducibility. This methodological framework is especially suited for detecting subtle, yet consistent, methylation changes associated with chronological aging across diverse cohorts, including variations by sex and health status. Our approach expands on prior frameworks^5^ by not only identifying DMPs, VMPs and entropy dynamics but also incorporating co-methylation network analyses (WGCNA), functional gene-level pathway meta-analysis (mitch), gene–gene pan-tissue correlation networks and in silico validations. Given the multilayer analytical framework employed, false discovery control was applied independently within each analysis layer using a stringent FDR threshold (FDR <0.005). Downstream interpretation prioritized signals that were consistent across tissues and analytical layers, providing an additional safeguard against spurious findings arising from multiple testing. No statistical methods were used to pre-determine sample sizes. Sample sizes reflect the availability of publicly accessible and collaborator-provided methylation datasets and are similar to or exceed those reported in previous cross-tissue epigenetic studies.

### Data collection and pre-processing

We assembled a large repository of DNAm profiles encompassing 15,995 human samples from 131 independent datasets, each assayed using Illumina methylation arrays (27k, 450k and EPIC). Data sources included 112 open-access studies from GEO, 5 from ArrayExpress, 2 from dbGaP, 1 from EGA and 10 from independent collaborators.

Datasets with fewer than ten samples or low age dispersion (standard deviation <5) were excluded to preserve statistical robustness. Samples from individuals diagnosed with cancer were removed to avoid confounding due to aberrant methylation patterns and individual samples with greater than 10% of probes failing detection (*P* > 0.01) were excluded during quality control. At the probe level, CpGs with missing *β* values, low bead count, non-CG content or cross-hybridization potential or mapping to single nucleotide polymorphisms or sex chromosomes in mixed-sex datasets were removed. No other datasets, samples or probes were excluded.

For datasets with available raw intensity data (Illumina Data Format), pre-processing and normalization were conducted in R using the ChAMP pipeline^48,49^. Normalization of type I and II probes was done via champ.norm, and unwanted technical variation (for example, slide and position effects) was adjusted within each dataset using ComBat^50^ when metadata was available. Sex mismatches between annotation and predicted sex were resolved using getSex from the minfi package.

For tissues with available high-confidence reference panels, cell-type proportions were estimated using EPISCORE^51^ and EpiDISH^52^ and included as covariates in downstream analyses to account for cellular heterogeneity. Additional unsupervised surrogate variable approaches were not applied, as reference-based methods have been shown to outperform surrogate variable approaches for controlling cell-type heterogeneity in DNAm studies^53^.

### Array platform heterogeneity and coverage bias

The included datasets were generated using Illumina 27k, 450k and EPIC methylation arrays, which differ in genomic CpG representation and CpG island enrichment (27k > 450k > EPIC). To minimize platform-driven biases, each dataset was pre-processed independently using the same standardized pipeline, and age-association models were fit within each study (including cohort-specific technical covariates where available). This design ensures that statistical inference is conducted within-platform and within-cohort, rather than relying on direct probe-level comparisons across arrays.

Downstream integration was performed at the level of summary statistics using meta-analysis, thereby combining effect estimates across independent cohorts while reducing sensitivity to platform-specific CpG coverage. For gene- and pathway-level analyses, CpG-level statistics were aggregated to gene-level measures before enrichment testing (including directionality-aware multi-contrast enrichment using mitch), further decreasing the likelihood that differences in CpG island probe density across arrays drive the biological interpretations. In addition, where tissue summaries combined studies from multiple platforms, results were interpreted as reflecting platform-robust associations supported across datasets rather than effects unique to any single array design.

### Differential methylation analysis (DMPs)

To identify age-associated DMPs, linear models were fitted independently within each dataset using limma^54^. Linear regression models assume normally distributed residuals. *M* values (logit-transformed *β* values) were used as the dependent variable to better approximate normality compared with raw *β* values. Formal normality testing was not performed on all datasets; data distribution was assumed to be approximately normal on the basis of the use of *M* values and the large sample sizes involved. Models incorporated available covariates such as sex, BMI and technical batch (Supplementary Table 1). Where applicable, repeated or related samples (for example, twins) were modeled using random effects via duplicateCorrelation. To decide on covariate inclusion in the models, we referenced GBD data and incorporated all non-age-related covariates, such as asthma, anemia, depression, etc. Age-related diseases such as Alzheimer’s, stroke, type 2 diabetes and cardiovascular disease were excluded. In addition, we included behavioral and aging-related modifiers as covariates, such as HIV, substance use and smoking. Analyses were based on *M* values (logit-transformed *β* values) to improve statistical properties.

For DMPs, summary statistics from each individual epigenome-wide association meta-analysis, segregated by tissue type, were first adjusted for bias and inflation using the empirical null distribution approach implemented in the ‘bacon’ package. The adjusted results were then meta-analyzed using an inverse-variance fixed-effects model in METAL, allowing for effect-size pooling across studies. Only CpG sites present in at least three datasets were retained in the meta-analysis to ensure reliability across platforms. Age-associated DMPs were identified using a stringent FDR threshold of <0.005.

To examine the directionality of age-related change (hypo- versus hypermethylation), we combined all datasets available for each tissue and defined methylation categories on the basis of the average methylation in young (<30 years) and old (>60 years) individuals. High methylation *β* ≥ 0.75, low methylation *β* ≤ 0.25 and intermediate *β* ≥ 0.25 and *β* ≤ 0.75 (remaining CpGs). This allowed the classification of DMPs according to methylation drift trajectories over time (for example, high-to-intermediate and intermediate-to-low).

### Variance analysis (VMPs)

To detect CpGs where the variance of DNAm changes systematically with age (VMPs), we employed a two-step Breusch–Pagan heteroscedasticity testing framework. Unlike standard linear regression, the Breusch–Pagan test does not assume homoscedasticity, making it appropriate for detecting age-associated variance shifts. This test evaluates whether the residual variance of DNAm is dependent on age, beyond what is explained by standard covariates.

Step 1: fit linear models to estimate residuals (DMPs analysis).

For each dataset, we first fitted a multivariate linear model for each CpG site, where the dependent variable was the *M* value (logit-transformed *β* value) of methylation at that CpG. The model was specified as

$${M}_{i}={\beta }_{0}+{\beta }_{1}\times \mathrm{Age}+{\beta }_{2}\times \mathrm{Sex}+{\beta }_{3}\times \mathrm{Disease}+\cdots +{\epsilon }_{i}.$$

${M}_{i}$ is the *M* value at CpG site *i*.

Covariates included: age (main variable of interest) as well as sex, BMI, technical batch and other dataset-specific covariates if available. Tissue type was not included as all analyses were stratified by tissue, and meta-analyses were performed within tissue type.

When datasets involved repeated measures (for example, twins and longitudinal data), a random effect was incorporated using duplicateCorrelation from the limma package^60^.

This regression model adjusts for known confounders and produces residuals—the component of variation in methylation not explained by age or other covariates.

Step 2: Breusch–Pagan test to assess variance change with age.

Next, for each CpG site, we squared the residuals from step 1 and used these as the dependent variable in a second univariate regression, where age was the sole predictor

$${\in }_{i}^{2}={\gamma }_{0}+{\gamma }_{1}\times\mathrm{Age}+\delta .$$

This models whether the variance of methylation residuals increases or decreases with age, that is, whether age explains variability in methylation, not just average level. CpGs where squared residuals were not normally distributed (Shapiro–Wilk test, *P* < 0.05) were excluded to avoid inflated type I error due to residual single nucleotide polymorphism effects or technical noise.

For VMPs, the output test statistics (*χ*^2^ values) from the Breusch–Pagan tests were meta-analyzed across datasets using a sample-size-weighted fixed-effects approach, also implemented in METAL. This method accounted for the differing sample sizes among datasets without requiring effect-size estimates. CpGs were included in the meta-analysis if they were present in at least 15% of all available samples, and significant age-associated VMPs were defined at an FDR <0.005.

### Cell-type-specific analysis

To then compare the effect of cell-type heterogeneity on age-related DNAm changes, we repeated all linear models, adjusting for cell-type proportions, in 11 tissues for which cell deconvolution panels were available (that is, via EPISCORE^51^ for all tissues except buccal, where EpiDISH^52^ was used). We then repeated the linear model for each dataset, including the cell types (except one, to avoid full collinearity), to remove the confounding effect of cell-type proportion on DMPs. A full panel of cell types included in each tissue have been reported by the EPISCORE package. In buccal tissue, we used the epidish function from the EpiDISH package to estimate the proportion of immune cells, fibroblasts and epithelial cells for each sample. Once again, we repeated each linear model and adjusted for all the largest cell-type proportions (except one, to avoid full collinearity) in buccal tissue.

Two examples of the linear models with cell-type adjustments.

1. Linear model for cell-type adjustments in brain tissue.
  $$\begin{array}{l}\mathrm{DNAm}\,\approx \,\beta \times \mathrm{age}\,+\,\beta \times \mathrm{sex}\,+\,\beta \times \mathrm{neuron}\,+\,\beta \times \mathrm{oligo}\\ \,+\,\beta \times \mathrm{astro}\,+\,\beta \times \mathrm{OPC}\,+\,\beta \times \mathrm{endo}\,+\,\beta \times \mathrm{microglia}\ldots \end{array}\,\,$$
2. Linear model for cell-type adjustments in buccal tissue.

$$\mathrm{DNAm}\,\approx \,\beta \times \mathrm{age}\,+\,\beta \times \mathrm{sex}\,+\,\beta \times \mathrm{fibroblasts}\,+\,\beta \times \mathrm{epithelial}\ldots$$

### Entropy analysis

To quantify methylation stochasticity and epigenetic complexity, Shannon entropy was calculated per sample across multiple sets of CpGs.

$$H=-\mathop{\sum }\limits_{i=1}^{N}[{\mathrm{MF}}_{i}\bullet {\log }_{2}\left({\mathrm{MF}}_{i}\right)+\left(1-{\mathrm{MF}}_{i}\right)\bullet {\log }_{2}\left(1-{\mathrm{MF}}_{i}\right)].$$

- MF_*i*_ is the methylation fraction (*β* value) of CpG *i*.
- Entropy ranges from 0 (fully methylated or unmethylated) to 1 (maximal uncertainty at ~50% methylation).

To isolate age-related effects, we conducted a multivariate linear model regressed *M* values against available covariates for each dataset (that is, sex, BMI and disease, etc., excluding age). We then added residuals to the mean signal to reconstruct adjusted *β* values. Entropy was then computed on these adjusted *β* matrices, preserving age effects while removing confounders. Age was then regressed against sample-level entropy in each dataset to assess age-dependency as follows:

$$\mathrm{Entropy}={\alpha }_{0}+{\alpha }_{1}\bullet \mathrm{Age}+\mathop{\sum }\limits_{j=2}^{n}{\alpha }_{j}\bullet {\mathrm{Covariate}}_{j}+\epsilon .$$

### Entropy was computed in a genome-wide manner across DMPs, VMPs and non-age-associated CpGs

For entropy, regression coefficients and standard errors from the linear models of age versus genome-wide methylation entropy (and other entropy subsets) were extracted for each dataset and meta-analyzed using a fixed-effects model implemented in the metafor package in R. Separate meta-analyses were performed for entropy calculated over all CpGs (genome-wide) as well as over specific subsets, including DMP-only, VMP-only, the intersection of DMPs and VMPs (DMP ∩ VMP) and CpGs not associated with age.

### Cross-tissue integration of DMPs and VMPs

To evaluate the extent of cross-tissue conservation of age-associated epigenetic signatures, we aggregated significant DMPs and VMPs across tissues into binary presence–absence matrices. Jaccard similarity indices and effect-size correlation matrices were computed to assess overlap and directional consistency of DMPs and VMPs across tissues. Shared CpGs were defined as those present in multiple tissues, and unique CpGs were analyzed for tissue-specific relevance. A combined similarity heat map, incorporating the Jaccard index (bottom triangle) and effect direction consistency (top triangle), was used to visualize tissue relationships. Permutation testing (10,000 iterations) was employed to determine whether observed DMP overlaps exceeded random expectations, and significance was denoted using empirical *P* values. Shared and unique DMP sets were subjected to GO enrichment analysis using missMethyl^61^. Additional integrative visualizations included effect-size matrix clustering and annotation of total and overlapping DMPs per tissue. These results enabled robust comparison of conservation, specificity and convergence of aging-related epigenetic signatures across tissues.

### Sensitivity analyses

To assess the influence of age range and sample characteristics on DMP and VMP discovery, we computed Pearson correlations between the number of significant hits and age distribution metrics (mean, median and range) per tissue. Tissues with broader age ranges exhibited higher DMP/VMP detection power, underscoring the importance of age variability in epigenetic studies. Scatter plots with regression and correlation statistics were generated to visualize these relationships. This sensitivity analysis confirmed that observed differences in DMP counts are partially attributable to differences in study design rather than purely biological variation.

### WGCNA

We implemented WGCNA on each tissue individually restricted to age-associated CpGs to identify modules of co-methylated CpGs. Before network construction, datasets were harmonized by aligning sample IDs between beta matrices and phenotype metadata, filtering out samples and CpGs with excessive missing values (>10% and >25%, respectively), and removing low-variance CpGs (lowest 20%). We followed the standard framework of WGCNA^55,56^. Initially, we determined the ideal soft-thresholding power for each tissue to uphold a scale-free topology in the methylation co-expression network The soft-thresholding power was selected on the basis of scale-free topology (*R*^2^ ≥ 0.8), where possible, and reasonable mean connectivity. Modules were identified using hierarchical clustering of topological overlap matrices with dynamic tree cutting, and their eigengenes were correlated with chronological age. Statistically significant modules (*P* < 0.05) were further analyzed^6^. Annotated CpGs within these modules were mapped to genes using the EPIC array annotation. GO enrichment was performed for each significant module using enrichGO (clusterProfiler). Modules were then classified on the basis of the direction of association with age (positive and negative), and their top hub CpGs and module-influential genes were extracted. Enriched GO terms and module–gene interactions were visualized using dot plots and network plots. Shared module-influential genes and their genomic contexts (for example, promoter, enhancer and gene body) were annotated, and chromatin state distributions were examined using Roadmap Epigenomics data.

### Identification of hub CpGs and module-influential genes

Weighted gene co-methylation networks were constructed using WGCNA, and age-associated modules were identified on the basis of significant correlations between module eigengenes and age (FDR-adjusted). Within each age-associated module, intramodular connectivity was quantified using kME, defined as the correlation between individual gene methylation profiles and the module eigengene. Gene significance for age was calculated as the correlation between gene-level methylation and chronological age.

Module-influential genes were defined as genes exhibiting both high intramodular connectivity (high absolute kME) and strong association with age within a given module. Genes were ranked within each module on the basis of these metrics, and the top-ranked genes were designated as module-influential genes. This approach prioritizes genes that are central to the module structure and most representative of the age-associated methylation signal. Module-influential gene identification is therefore network-based and does not constitute an additional differential methylation test.

### Gene-level functional meta-analysis

DMP-level *t*-statistics were aggregated to the gene level using a sum-of-*t*-statistics approach, with CpGs mapping to multiple genes parsed and split accordingly. This produced a gene × tissue matrix of signed effect sizes, which was used as input for multivariate pathway enrichment analysis using the mitch framework.

mitch was selected because it enables multi-contrast, directionality-aware pathway enrichment without relying on CpG- or gene-level significance thresholds, making it well suited for cross-tissue analyses with heterogeneous sample sizes and statistical power. By jointly modeling coordinated shifts in effect direction across tissues, mitch can detect biologically meaningful pathway-level signals even when individual features do not reach statistical significance in underpowered tissues. Gene sets were obtained from MSigDB (GO Biological Process), and enrichment was tested using multivariate analysis of variance-based multivariate models. Pathways showing significant coordinated enrichment across tissues (FDR <0.005) were further classified by the direction and consistency of effects (for example, ‘s.dist’).

### Pan-tissue gene–gene correlation network analysis

The mitch-derived *t*-statistic matrix was *z*-score transformed, filtered for genes with low missingness and converted into a pairwise Spearman correlation matrix. Hierarchical clustering (average linkage) was used to generate gene dendrograms, followed by static (*k* = 10 and *k* = 30) and adaptive dynamic tree cutting (deepSplit 2, minClusterSize 50) to define gene modules. Directional consistency across tissues was determined for each module (for example, universal gain/loss, tissue-specific and divergent), and average *t*-statistics per module–tissue combination were visualized via heat maps. Top hub genes (highest connectivity) and top module-influential genes (highest combined connectivity × average *t*-statistic) were identified for each module. Gene set enrichment for each module was performed using clusterProfiler with MSigDB terms.

### In silico robustness simulations

We developed an in silico perturbation framework to quantify the impact of methylation shifts in individual genes on global module structure. Perturbations were simulated by zeroing out (knockdown) or doubling (overexpression) *t*-statistic values across tissues. For each gene perturbation, average module activity was recalculated and compared with baseline. Modulation was quantified as the summed absolute difference in module activity across tissues, and genes ranking highest on this metric were prioritized for further interpretation. Simulations were also run for combinations of genes (for example, the PCDHG family). Each module’s modulation profile was annotated for pathway enrichment and classified directionally on the basis of whether the perturbed module was enriched in age- or disease-associated pathways, with modulations interpreted as potentially beneficial or detrimental accordingly. Genes showing consistent high perturbation scores across multiple modules and tissues were noted. Fragile modules were defined as modules in which perturbation of individual genes resulted in large shifts in module eigengene values and pathway enrichment profiles, indicating high sensitivity to epigenetic modulation. Conversely, resilient modules were defined as modules that retained stable eigengene structure and functional enrichment following comparable perturbations, suggesting robustness to epigenetic change.

### Reporting summary

Further information on research design is available in the Nature Portfolio Reporting Summary linked to this article.

## Supplementary information

Supplementary Information
Supplementary Fig. 1 and Tables 1–9.

Reporting Summary

Peer Review File

Supplementary Table 1
Summary of datasets included in the studies and models used for each dataset.
Supplementary Table 2
Global methylation analysis results.
Supplementary Table 3
DMPs and VMPs before and after cell-type correction.
Supplementary Table 4
Power calculation results.
Supplementary Table 5
Permutation-based sensitivity analysis for the overlap likelihood of VMPs and DMPs.
Supplementary Table 6
Pan-tissue analysis of gene–gene networks.
Supplementary Table 7
In silico perturbation analysis findings featuring key contributing genes and pathways.
Supplementary Table 8
Transitions in CpG methylation states between younger (≤30 years) and older (≥60 years) individuals across tissues. CpGs are categorized as low (<0.25), intermediate (0.25–0.75) or high (>0.75), and values represent the number (or proportion) shifting between states with age. Transitions toward intermediate methylation (low → intermediate and high → intermediate) are consistent with stochastic/entropic drift, whereas maintenance within or shifts reinforcing extreme states may reflect more structured remodeling. Interpretation should be made alongside direction-of-change results (Fig. 4), as apparent hypermethylation can arise from drift toward intermediate states when baseline methylation is predominantly low.
Supplementary Table 9
Top pan-tissue DMPs present in 13 or more tissues.

## Data Availability

The DNAm datasets analyzed in this study were obtained from publicly accessible repositories and are available via the Gene Expression Omnibus at https://www.ncbi.nlm.nih.gov/geo/, via ArrayExpress at (https://www.ebi.ac.uk/arrayexpress/), via dbGaP at https://www.ncbi.nlm.nih.gov/gap/ and via the European Genome–Phenome Archive at https://ega-archive.org/. Accession numbers and access details for all 131 datasets are provided in Supplementary Table 1. The ten datasets obtained from independent collaborators are available upon request from the corresponding author N.E. (nir.eynon@monash.edu). Summary statistics and processed data generated in this study are available via the Human Aging Methylation Atlas at https://bioinformatics.erc.monash.edu/apps/human-aging-atlas/.

## Code availability

Custom code used for data processing, differential methylation analysis, variance analysis, entropy calculation, WGCNA and in silico perturbation simulations is available via GitHub at https://github.com/MacsueJacques/DNA-Methylation-Aging-Atlas-Across-17-Human-Tissues. All third-party software packages used are cited in Methods and listed in the Reporting Summary with version numbers.
