# Supplementary material for: Meta-analysis of DNA methylation aging signatures in 17 human tissues
Source: Nat Aging. 2026 Jun 26;6(7):1501–15. doi: 10.1038/s43587-026-01164-5 (PMC13375584; doi:10.1038/s43587-026-01164-5)
Supplement: Supplementary file 1 — Supplementary Information Supplementary Fig. 1 and Tables 1–9. [file 43587_2026_1164_MOESM1_ESM.pdf]

nature aging

Supplementary information https://doi.org/10.1038/s43587-026-01164-5

Meta-analysis of DNA methylation aging signatures in 17 human tissues

In the format provided by the authors and unedited

Meta Analysis of DNA Methylation Aging Signatures in 17 Human Tissues

Macsue Jacques[1], Kirsten Seale[2,3], Sarah Voisin[4], Anna Lysenko[1], Robin Grolaux[1], Bernadette Jones-Freeman[1], Severine Lamon[5], Mandhiri Dushyanthi Abeysooria[1], Itamar Levinger[3,6], Carlie Bauer[3], Adam P. Sharples[7], Aino Heikkinen[8,9], Elina Sillanpaa[10,11], Miina Ollikainen[8,9], Cassandra Smith[12,13], James R. Broatch[3], Navabeh Zarekookandeh[3], Linn Gillberg[14,15], Ida Blom[14,15], Jesse R. Poganik[16], Mahdi Moqri[16], Vadim N. Gladyshev[16], Matthew Taper[17,18], Cassandra Malecki[17,18], Sean Lal[17,18], Nathalie Saurat[1], Steve Horvath[19], Andrew Teschendorff[20], Nir Eynon[1*]

Affiliations

1. Australian Regenerative Medicine Institute, Monash University, Clayton, Victoria, Australia.

2. TruDiagnostics, Lexington, KY, USA

3. Institute for Health and Sport, Victoria University, Footscray, VIC, Australia

4. Novo Nordisk, Copenhagen, Denamark

5. Institute for Physical Activity and Nutrition, School of Exercise and Nutrition Sciences, Deakin University, Geelong, Australia

6. Australian Institute for Musculoskeletal Science (AIMSS), Victoria University and Western Health, St Albans, Australia

7. Institute for Physical Performance, Norwegian School of Sport Sciences, Olso, Norway.

8. Minerva Foundation Institute for Medical Research, Helsinki, Finland

9. Institute for Molecular Medicine Finland (FIMM), HiLIFE, University of Helsinki, Helsinki, Finland

10. Faculty of Sport and Health Sciences, University of Jyväskylä, Jyväskylä, Finland

11. Wellbeing Services County of Central Finland, Jyväskylä, Finland

12. Nutrition & Health Innovation Research Institute, School of Medical and Health Sciences, Edith Cowan University, Perth, Western Australia, Australia

13. Medical School, The University of Western Australia, Perth, Western Australia, Australia

14. Department of Biomedical Sciences, University of Copenhagen, Copenhagen, Denmark

15. Department of Biomedical Sciences, Faculty of Health and Medical Sciences, University of Copenhagen, Copenhagen, Denmark

16. Division of Genetics, Department of Medicine, Brigham and Women's Hospital, Harvard Medical School, Boston, MA, USA

17. Faculty of Medicine and Health, The University of Sydney & Royal Prince Alfred Hospital

18. The Baird Institute for Applied Lung and Heart Research, Sydney, Australia

19. Altos Labs, Cambridge, United Kingdom

20. Shanghai Institute of Nutrition and Health, Chinese Academy of Sciences, University of Chinese Academy of Sciences, Shanghai, China.

***Correspondence:** Nir Eynon, PhD, Australian Regenerative Medicine Institute, 15 Innovation walk, Clayton, VIC 3800, Faculty of Medicine, Nursing & Health Sciences, Monash University, Australia: Email: Nir.Eynon@monash.edu

Supplementary Figures:

Figure 1 Overview of the analytical framework applied in this study

Supplementary Tables:

Table 1 Summary of datasets included in the studies and models used for each dataset.

Table 2 Global Methylation analysis results

Table 3 DMPs and VMPs before and after cell-type correction

Table 4 Power calculation results

Table 5 Permutation-based sensitivity analysis for the overlap likelihood of VMPs and DMPs.

Table 6 Pan-tissue analysis of gene-gene networks

Table 7 In-silico perturbation analysis findings featuring key contributing genes and pathways

Table 8 Transitions in CpG methylation states between younger (≤30 years) and older (≥60 years) individuals across tissues. CpGs are categorized as low (<0.25), intermediate (0.25–0.75), or high (>0.75), and values represent the number (or proportion) shifting between states with age. Transitions toward intermediate methylation (low → intermediate; high → intermediate) are consistent with stochastic/entropic drift, whereas maintenance within or shifts reinforcing extreme states may reflect more structured remodeling. Interpretation should be made alongside direction-of-change results (Fig 4), as apparent hypermethylation can arise from drift toward intermediate states when baseline methylation is predominantly low.

Table 9 Top pan-tissue DMPs present in 13 or more tissues.

Supplementary Figures

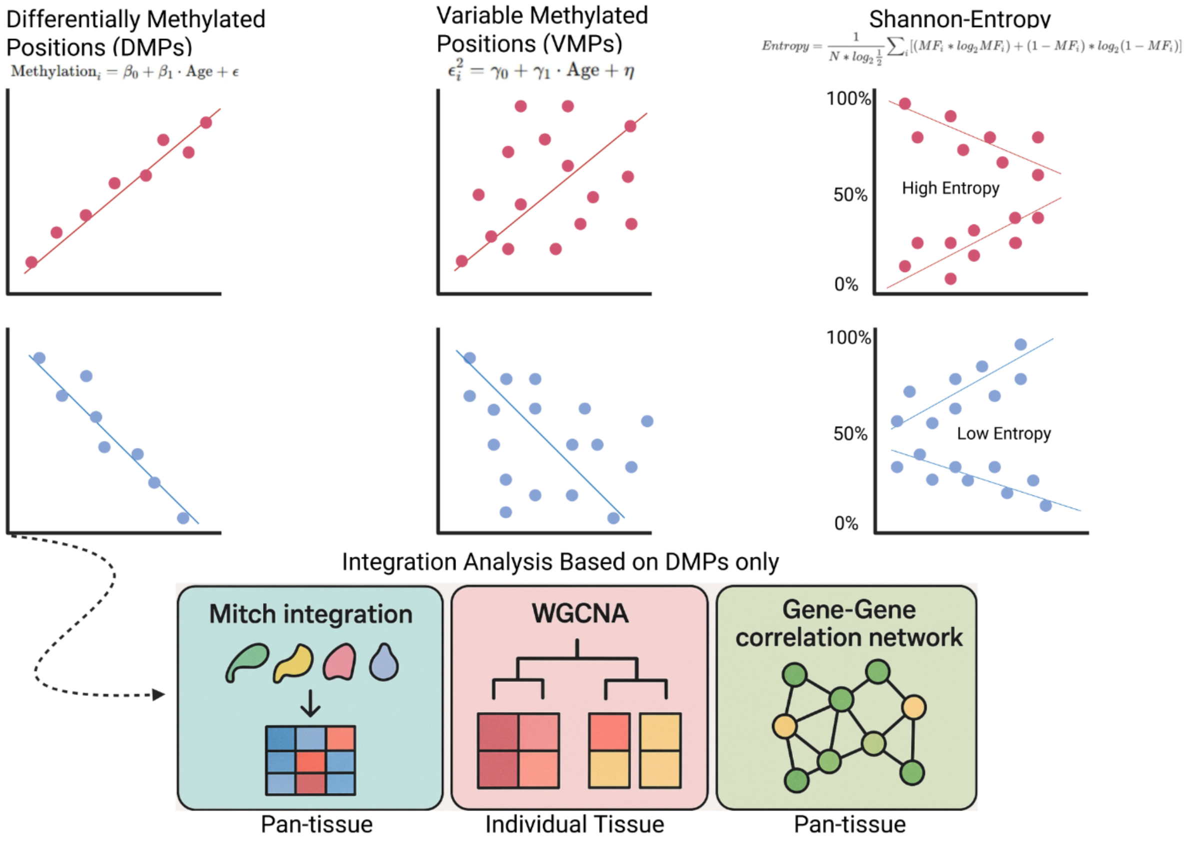

Supplementary Figure 1 Overview of the analytical framework applied in this study. Illustration of the three methylation metrics used to characterise age-associated epigenetic changes across tissues. Differentially methylated positions (DMPs) are identified using a linear regression model, capturing directional shifts in mean methylation level with age; red dots and regression line indicate hypermethylation with age and blue dots and regression line indicate hypomethylation with age. Variably methylated positions (VMPs) are identified using a heteroscedasticity-based framework, detecting age-associated increases in inter-individual variance in methylation; red and blue indicate increasing and decreasing variance with age respectively. Shannon entropy is calculated per sample using the formula shown, quantifying genome-wide methylation disorder; high entropy (red) reflects methylation values converging toward 50%, indicating maximum uncertainty, while low entropy (blue) reflects methylation values approaching 0% or 100%. The lower panel illustrates the three downstream integration analyses applied to DMP-level results: mitch integration, a pan-tissue multivariate pathway enrichment approach combining gene-level t-statistics across tissues into a gene × tissue matrix; Weighted Gene Co-expression Network Analysis (WGCNA), applied at the individual tissue level to identify co-methylation modules; and gene–gene correlation network analysis, a pan-tissue approach constructing pairwise Spearman correlation matrices from tissue-level t-statistics to identify genes with coordinated aging trajectories across organs.
