# Supplementary material for: Meta-analysis of DNA methylation aging signatures in 17 human tissues
Source: Nat Aging. 2026 Jun 26;6(7):1501–15. doi: 10.1038/s43587-026-01164-5 (PMC13375584; doi:10.1038/s43587-026-01164-5)
Supplement: Supplementary file 2 — Reporting Summary [file 43587_2026_1164_MOESM2_ESM.pdf]

Corresponding author(s): Nir Eynon

Last updated by author(s): May 4, 2026

Reporting Summary

Nature Portfolio wishes to improve the reproducibility of the work that we publish. This form provides structure for consistency and transparency in reporting. For further information on Nature Portfolio policies, see our Editorial Policies and the Editorial Policy Checklist.

Statistics

For all statistical analyses, confirm that the following items are present in the figure legend, table legend, main text, or Methods section.

n/a	Confirmed	
☐	☒	The exact sample size (*n*) for each experimental group/condition, given as a discrete number and unit of measurement
☐	☒	A statement on whether measurements were taken from distinct samples or whether the same sample was measured repeatedly
☐	☒	The statistical test(s) used AND whether they are one- or two-sided<br>*Only common tests should be described solely by name; describe more complex techniques in the Methods section.*
☐	☒	A description of all covariates tested
☐	☒	A description of any assumptions or corrections, such as tests of normality and adjustment for multiple comparisons
☐	☒	A full description of the statistical parameters including central tendency (e.g. means) or other basic estimates (e.g. regression coefficient) AND variation (e.g. standard deviation) or associated estimates of uncertainty (e.g. confidence intervals)
☐	☒	For null hypothesis testing, the test statistic (e.g. *F*, *t*, *r*) with confidence intervals, effect sizes, degrees of freedom and *P* value noted<br>*Give P values as exact values whenever suitable.*
☒	☐	For Bayesian analysis, information on the choice of priors and Markov chain Monte Carlo settings
☐	☒	For hierarchical and complex designs, identification of the appropriate level for tests and full reporting of outcomes
☐	☒	Estimates of effect sizes (e.g. Cohen's *d*, Pearson's *r*), indicating how they were calculated

Our web collection on statistics for biologists contains articles on many of the points above.

Software and code

Policy information about availability of computer code

Data collection	Data were collected from public and controlled-access repositories using open-source tools and repository-specific download procedures. GEO datasets were retrieved using the GEOquery R package (v2.74.0). Additional datasets were obtained from secure shared folders/data-transfer links provided by collaborators.
Data analysis	Data analysis was performed using R version 4.4.2 and open-source R/Bioconductor and CRAN packages, including limma v3.62.2, ChAMP v2.36.0, minfi v1.52.1, sva v3.54.0, EpiSCORE v0.9.5, EpiDISH v2.22.0, bacon v1.34.0, WGCNA v1.73, mitch v1.18.4, clusterProfiler v4.14.6, metafor v4.6-0 and missMethyl v1.40.3. Meta-analysis was performed using METAL v2011-03-25. Custom R scripts were used for dataset harmonisation, covariate processing, EWAS, VMP, entropy, WGCNA, cross-tissue integration, visualisation and in-silico perturbation analyses. No commercial software was used for statistical analysis.

For manuscripts utilizing custom algorithms or software that are central to the research but not yet described in published literature, software must be made available to editors and reviewers. We strongly encourage code deposition in a community repository (e.g. GitHub). See the Nature Portfolio guidelines for submitting code & software for further information.

Data

Policy information about availability of data

All manuscripts must include a data availability statement. This statement should provide the following information, where applicable:

- Accession codes, unique identifiers, or web links for publicly available datasets
- A description of any restrictions on data availability
- For clinical datasets or third party data, please ensure that the statement adheres to our policy

DNA methylation datasets analysed in this study were obtained from publicly available repositories and collaborator-provided datasets. Publicly available datasets can be accessed through their original repositories using the accession identifiers provided in Supplementary Table 1. Collaborator-provided datasets are not publicly redistributed by the authors because of data-sharing restrictions; hence, access to private datasets should be requested from the original data custodians.

Research involving human participants, their data, or biological material

Policy information about studies with human participants or human data. See also policy information about sex, gender (identity/presentation), and sexual orientation and race, ethnicity and racism.

Reporting on sex and gender	Sex was included as a covariate in all linear regression models where data were available. No analyses were stratified by sex or gender. Gender identity and sexual orientation data were not collected or analysed in this study.
Reporting on race, ethnicity, or other socially relevant groupings	Race, ethnicity, and other socially relevant groupings were not systematically collected or reported across the 131 datasets included in this meta-analysis. As a secondary analysis of publicly available and collaborator-provided datasets, the demographic composition of individual cohorts reflects the original study designs and is detailed where available in Supplementary Table 1. The findings should be interpreted with consideration of potential population-level differences in DNA methylation patterns across ancestries.
Population characteristics	This study analysed DNA methylation data from 15,995 human samples spanning 17 tissue types, obtained from 131 independent datasets. Samples were derived from adult individuals across a broad age range. Detailed information on sample sizes, age ranges, sex distribution, and cohort characteristics for each dataset is provided in Supplementary Table 1. Samples from individuals with a cancer diagnosis were excluded from all analyses.
Recruitment	This study is a secondary analysis of existing publicly available and collaborator-provided datasets. No new participants were recruited by the authors. Participant recruitment for each contributing dataset was conducted by the original study authors according to their respective study protocols, as described in the original publications referenced in Supplementary Table 1.
Ethics oversight	This study constitutes a secondary analysis of publicly available and collaborator-shared data and did not require additional ethical approval. All original studies were conducted in accordance with the Declaration of Helsinki. Each contributing dataset was collected under ethical approval from the relevant institutional review board or ethics committee at the originating institution, and written informed consent was obtained from all participants in the original studies. For datasets collected by the authors' institutions, ethical approval was granted by the Human Research Ethics Committee of Victoria University

Note that full information on the approval of the study protocol must also be provided in the manuscript.

Field-specific reporting

Please select the one below that is the best fit for your research. If you are not sure, read the appropriate sections before making your selection.

☒ Life sciences ☐ Behavioural & social sciences ☐ Ecological, evolutionary & environmental sciences

For a reference copy of the document with all sections, see nature.com/documents/nr-reporting-summary-flat.pdf

Life sciences study design

All studies must disclose on these points even when the disclosure is negative.

Sample size	No statistical methods were used to pre-determine sample sizes. Sample sizes reflect the availability of publicly accessible and collaborator-provided DNA methylation datasets at the time of analysis, and are similar to or exceed those reported in previous cross-tissue epigenetic studies (refs 4, 5, 19). A total of 15,995 human samples from 131 independent datasets across 17 tissues were included. Per-tissue sample sizes are provided in Supplementary Table 1. Power analyses were performed post-hoc for each tissue to assess detectability of effect sizes at FDR ≤ 0.005; results are reported in Supplementary Table 4.
Data exclusions	Datasets with fewer than 10 samples or low age dispersion (standard deviation < 5 years) were excluded to preserve statistical robustness. Samples from individuals diagnosed with cancer were removed to avoid confounding due to aberrant methylation patterns. Individual samples with greater than 10% of probes failing detection ($p > 0.01$) were excluded during quality control. At the probe level, CpGs with missing β-values, low bead count, non-CG content, cross-hybridisation potential, or mapping to SNPs or sex chromosomes in mixed-sex datasets were removed. No other datasets, samples, or probes were excluded.
Replication	This study is a computational meta-analysis of existing datasets. Replication was addressed through the meta-analytic framework itself — age-associated signals were required to be consistent across multiple independent datasets within each tissue before being considered robust.

Permutation testing was used to confirm that cross-tissue overlaps of DMPs and VMPs exceeded chance expectations (Supplementary Table 5). The universal hypermethylation module containing PCDHGA1 as the top module-influential gene was identified consistently across all tissues and analytical layers, providing internal replication of this finding. All code and data are publicly available to enable independent replication by other researchers.

Randomization

Randomization was not applicable to this study. This is a secondary analysis of existing observational datasets in which samples were not allocated into experimental groups by the authors. Datasets were assigned to tissue categories based on the tissue of origin as annotated in the original studies. Potential confounding factors were controlled statistically through inclusion of relevant covariates in all linear regression models, including sex, BMI, technical batch, and disease-related covariates where available.

Blinding

Blinding was not applicable to this study. This is a computational meta-analysis of existing datasets with no experimental group allocation performed by the authors. All analyses were conducted using pre-specified analytical pipelines applied systematically across all tissues and datasets. No subjective outcome assessment was involved in the primary analyses.

Reporting for specific materials, systems and methods

We require information from authors about some types of materials, experimental systems and methods used in many studies. Here, indicate whether each material, system or method listed is relevant to your study. If you are not sure if a list item applies to your research, read the appropriate section before selecting a response.

Materials & experimental systems

n/a	Involved in the study
☒	☐ Antibodies
☒	☐ Eukaryotic cell lines
☒	☐ Palaeontology and archaeology
☒	☐ Animals and other organisms
☒	☐ Clinical data
☒	☐ Dual use research of concern
☒	☐ Plants

Methods

n/a	Involved in the study
☒	☐ ChIP-seq
☒	☐ Flow cytometry
☒	☐ MRI-based neuroimaging

Plants

Seed stocks

Report on the source of all seed stocks or other plant material used. If applicable, state the seed stock centre and catalogue number. If plant specimens were collected from the field, describe the collection location, date and sampling procedures.

Novel plant genotypes

Describe the methods by which all novel plant genotypes were produced. This includes those generated by transgenic approaches, gene editing, chemical/radiation-based mutagenesis and hybridization. For transgenic lines, describe the transformation method, the number of independent lines analyzed and the generation upon which experiments were performed. For gene-edited lines, describe the editor used, the endogenous sequence targeted for editing, the targeting guide RNA sequence (if applicable) and how the editor was applied.

Authentication

Describe any authentication procedures for each seed stock used or novel genotype generated. Describe any experiments used to assess the effect of a mutation and, where applicable, how potential secondary effects (e.g. second site T-DNA insertions, mosiacism, off-target gene editing) were examined.
