# Supplementary material for: Meta-analysis of DNA methylation aging signatures in 17 human tissues
Source: Nat Aging. 2026 Jun 26;6(7):1501–15. doi: 10.1038/s43587-026-01164-5 (PMC13375584; doi:10.1038/s43587-026-01164-5)
Supplement: Supplementary file 3 — Peer Review File [file 43587_2026_1164_MOESM3_ESM.pdf]

nature portfolio

Peer Review File

Meta Analysis of DNA Methylation Aging Signatures in 17 Human Tissues

Corresponding Author: Professor NIR EYNON

Version 0:

Reviewer comments:

Reviewer #1

(Remarks to the Author)
This manuscript presents a comprehensive DNA methylation aging atlas across 17 human tissues, analyzing over 15,000 methylomes through multidimensional approaches including DMPs, VMPs, and Shannon entropy. The study identifies both tissue-specific and conserved epigenetic aging signatures, with network analyses revealing key driver genes and pathways. The authors provide a valuable open-access resource for investigating molecular aging mechanisms. This study represents a significant contribution to the epigenetics of aging field. I have following suggestions to improve the paper.

1. The substantial disparity in sample sizes across tissues (e.g., limited samples for prostate, rectum, and stomach) raises concerns about detection power and comparative reliability. The authors should discuss how this imbalance affects cross-tissue comparisons and whether statistical corrections were adequately applied.

2. The use of bulk tissue data without proper cell-type composition adjustment may confound observed methylation patterns. Age-related changes in cell population proportions could mimic epigenetic aging signals. Cellular deconvolution analysis or detailed discussion of this limitation is warranted.

3. The exclusive reliance on cross-sectional data prevents distinction between true aging effects and cohort differences. Longitudinal validations of identified epigenetic signatures are critical and would significantly strengthen the findings.

4. While bioinformatic analyses are comprehensive, experimental validation of key discoveries (particularly the proposed central role of PCDHG gene family) is missing. Integration with expression data or functional assays would enhance mechanistic insights.

5. The combination of 131 datasets from different platforms and batches, despite statistical corrections, requires more rigorous demonstration of data harmonization efficacy and consistency across technical variations.

6. The multilayer analytical approach (DMPs, VMPs, entropy, WGCNA, mitch) creates substantial multiple testing burden. Clearer description of overall false discovery rate control strategies is needed.

7. The potential implications for biomarkers and therapeutic targets require more substantive discussion regarding practical applications in age-related disease prediction and intervention strategies.

Minor points
1. Key terms like "fragile modules" and "resilient modules" require more precise definitions, especially when discussing their functional significance.

2. Some conclusions appear overly definitive for correlative findings (e.g., definitive statements about PCDHG gene family roles). More cautious language reflecting the observational nature of the study is appropriate.

3. While methods are generally detailed, certain parameter selections (e.g., WGCNA soft-thresholding criteria) need clearer justification.

(Remarks on code availability)
It is adequate

Reviewer #2

(Remarks to the Author)
Jacques et al. have submitted a Resource manuscript to Nature Aging interrogating ageing-related DNA methylation (DNAm) changes in ~15k DNAm array samples across 17 tissues. The tissue sets comprising this meta-analysis range in size from ~7k samples for brain-derived to 73 from the Pancreas. However, it importantly excluded the much larger blood-derived datasets available in order not to be heavily biased by that well-analysed tissue type.

The authors analysed this dataset for DMPs, VMPs, and Shannon entropy. Interestingly, the authors identified through network analysis and shared signatures, genes and gene families with cross-tissue driver effects, such as Protocadherin gamma, HOX, and Histone Deacetylase 4. These genes and pathways highlight candidates for further follow-up work. Additionally, they state they provide these data as a browsable resource for the community.
This work is novel and of broad interest to a wide audience - as analysis of the ageing DNA methylome is a very active area of research, and most previous work has been biased towards blood-derived results. However, some of the interpretations made in the manuscript could be more nuanced. My comments for the authors to consider are listed below

Major

1. The R shiny application link was not working at my time of access.

2. The authors acknowledge the major issue of cell type heterogeneity within these tissues – however, this needs to be considered regarding many of the statements about tissue-specific differences. For example, it may be that tissues showing strong intermediate methylation signals are due to a predominant mixture of an unmethylated cell-type/s and a methylated cell type/s. These are observed to start at an intermediate level and that rises as the unmethylated cell/s hypermethylated with age. This should be considered along with statements about 'progressive chromatin condensation and regional silencing as a potential aging mechanism'. Also, regarding the statement that 'skeletal muscle and lung tissues exhibited 61% and 50%, respectively, of DMPs in the intermediate methylation state'.

3. Regarding the Methodology – could the authors comment on whether they considered using unsupervised reference-free methods to attempt to capture cell-type effects (SVA etc)?

4. The authors used 27k, 450k, and EPIC DNAm – all DNAm arrays are biased compared to the genomic distribution of CpGs. However, the arrays have variation in this bias, such as differing enrichment of CpG island regions – with the 27k array being predominantly CpGs in dense CpG regions. It may have been factored in, but it was not clear to me whether the differing proportions of array type in the different tissues were accounted for - i.e., more contributed to by 27k (e.g., Prostate is one third 27k). If not, this may have influenced the tissue summary results – e.g. greater proportion of CpG in CpG islands. Did CpG island array bias (27k > 450k > EPIC) contribute to any of the signals driving 'broad upregulation of pathways tied to transcriptional regulation'?

5. The authors stated that '65% of DMPs in the young subjects were from low methylation regions'. They should also explore this further by defining the CpG% density (as well as island/shore classification) of these defined low methylation regions. Thus, they could add further details to the statement that in 'most tissues, age-DMPs were primarily found in regions with low methylation (<25%)'.

6. The VMP analysis showed minimal consistent findings – was there any evidence that these CpGs are more confounded genetically – i.e. were they enriched for being CpGs under mQTL effects? [1].

7. The authors acknowledge the likely reduced ability to detect DMPs in tissues with limited samples, but did they perform specific power calculations for each tissue [2] (also taking into consideration the array type contributions to each tissue set) that could then be used to inform on the results observed?

8. Blood infiltration/contamination of tissue samples is another issue that could be assessed by cellular deconvolution.

Minor

1. The authors state 'hemi-methylated' (line 247) – which specifically refers to DNAm on only one of the two strands is methylated [3] – are the authors instead referring to intermediately methylated loci?

2. Pearson capitalised (line 739).

1. Oliva, M., et al., DNA methylation QTL mapping across diverse human tissues provides molecular links between genetic variation and complex traits. Nat Genet, 2023. 55(1): p. 112-122.
2. Mansell, G., et al., Guidance for DNA methylation studies: statistical insights from the Illumina EPIC array. BMC Genomics, 2019. 20(1): p. 366.
3. Sharif, J. and H. Koseki, Hemimethylation: DNA's lasting odd couple. Science, 2018. 359(6380): p. 1102-1103.

(Remarks on code availability)

Reviewer #3

(Remarks to the Author)
Overall, this is a statistically robust study which, by integrating many datasets, could serve as a reference to understand tissue specific methylation trajectories. As the authors write, there has been limited research into age-related methylation change outside of select tissues. Their study addresses this gap in the literature.

Major concerns

The major problem with this study is that it suffers from profound issues in the communication of findings, related to the text and figures. Textually, the manuscript is quite verbose and repetitive and should be greatly abbreviated. Additionally, many conclusions are interpreted as causal or relevant to aging as a whole, when they are purely correlative and relevant only to epigenetic aging specifically. The main weaknesses of the figures are that (1) they are not conducive to comparing the results across tissues, despite this being the main focus of the study, and (2) are poorly formatted in terms of the text size, legend, and color. So, while the methods and study design are sound, the presentation and communication of the results prevent this manuscript from being useful.

Specific points

A figure panel which makes it easy to compare the results across tissues is necessary for the presentation of the results in Figures 1, 2, 3, and 4. Suggestions for figures 2 and 4 follow, but this should be applied to all four of these figures. The study is meant to facilitate comparison of methylomic changes across tissues and so this is essential.

Figure 2. The pie charts are aesthetically pleasing but not conducive to cross-tissue comparisons. The results from this figure would be clearer if presented in another form. I leave it to the authors to decide what that should be. One option would be a bar graph where tissues are arranged along the x-axis, each tissue has 3 bars whose height denotes the count of CpGs which begin lowly, intermediately, or highly methylated, and each of these bars is a "stacked" bar, where one color within the bar shows the proportion of CpGs which gain methylation and another color shows the proportion which lose methylation (within each starting-methylation class). In the new rendition of this figure, color should be used more thoughtfully to add interpretability instead of the current state where the colors chosen appear to be random, yet are re-used across panels.

Figure 4. A figure which shows the effect size of entropy gain/loss in each tissue should be added to allow comparison across tissues. This could be a barplot where for each tissue there are 4 bars the height of each corresponds to the slope of entropy gain/loss for that class of CpG sites in that tissue. The statistical of this slope should be reported along with the effect size (in the figure).

For DMPs and VMPs, results are presented across all datasets for a tissue. Why then for entropy are the results only from the largest dataset from each tissue? The analysis of the association between entropy and age should be re-done using all datasets for consistency.

Why were skeletal muscle, adipose tissue, blood, and brain chosen for WCGNA? Did these tissues have the greatest number of samples? Either a quantitative metric should be used to justify the choice of which tissues to do WCGNA on (such as number of samples) or results from all tissues should be shown.

The authors conclude that "that aging is not solely characterized by stochastic drift" because they find age-related methylation changes which are (1) tissue-specific, (2) directional, and (3) are enriched in certain pathways. To make such a statement, the authors should show that a stochastic model cannot recover such patterns. Results from studies such as Myer et al. 2025 persuasively make the opposite claim. One could imagine that early in life, different tissues start with different methylomic patterns from which point stochastic drift accounts for all epigenetic change. Perhaps, this model could explain all three observations mentioned above in which case aging would be solely characterized by stochastic drift. Generally, highly confident statements regarding the source or reason of methylomic change should be removed or supported with further analysis.

Throughout, the authors make overly strong statements based on purely correlative analyses. For example, “Key disruptors, including PCDHGA1, MEST, HDAC4, and HOX genes, exacerbated aging signals across tissues.” These genes are not causally validated "disruptors of aging signals" they simply changed the WCGNA clustering when simulated to have different methylation values. Relatedly, the terminology of "driver gene," used in Figure 5 and onward, should be changed because "drive" implies causation, of which none has been shown. Numerous statements such as this should be clarified and toned-down.

Many statements imply that the results of this study reflect characteristics of aging as a whole. E.g., “Additionally, using advanced computational tools, we have identified key drivers of ageing in different tissues and uncovered unique ageing pathways and coordinated methylation patterns among tissues." This is inaccurate. This study only looked at *epigenetic aging* which may or may not relate to other aspects of aging writ large. This should be clarified throughout.

The text is very long and repetitive. For example, DMP, VMP, and entropy are defined and explained multiple times. The authors should consider how they can massively streamline the manuscript. Suggestions:

The first results section can be almost entirely eliminated without loss of information

Figure captions can be made shorter and more precise

The discussion is far too long and should be shortened to more succinctly and elegantly integrate and discuss the findings of the study. For example, "Recent research links hypermethylation in the PCDHG family to reduced white matter in the brain, a marker of accelerated cognitive decline. This connection is particularly intriguing since previous studies have shown that abdominal fat can predict decreases in cerebral white matter" how is the fact that abdominal fat predicts decreases in cerebral white matter related whatsoever to the findings of this study? There are also multiple epically run-on sentences such as from line 530-535.

The figure legends, axes tick labels, axes labels, and much of the text in figures is too small and low-resolution to read. Nearly every panel of every figure must be re-formatted to achieve legibility. Colors should be used more thoughtfully (e.g., diverging color maps should not be used for monotonic data).

The information in Figure 3 would better be represented as a volcano plot, where the x-axis is the coefficient relating age and the squared residual from `$\in i2= \gamma 0 + \gamma 1 \cdot Age + \delta$` and the y-axis is the log10 p value for this coefficient.

It is unclear which, and how many, covariates were adjusted for in the DMP and VMP analyses. A list of the covariates adjusted for in each study should be presented. If the same set of covariates was not adjusted for across all studies, the authors should justify this inconsistency or use a set of covariates available in all studies.

It is unclear to me what Figure 5B/D/F/H show. How is a gene determined to be a "top gene driving enrichment in age-associated modules"? The methods section merely says "Modules ... top hub CpGs and driver genes were extracted” but does not specify how this was done. What metric was used to identify driver genes? How was statistical significance assessed? A statistical significance cut-off with multiple testing correction should be used to ensure the “driver genes” are meaningful. This is especially important because of the authors’ focus on the frequency of PCDHG genes being found to be "driver genes” across tissues.

(Remarks on code availability)

Version 1:

Reviewer comments:

Reviewer #1

(Remarks to the Author)
The authors have submitted a substantially revised manuscript, alongside a very thorough and thoughtful rebuttal. Constructing an epigenetic aging atlas across 17 human tissues utilizing over 15,000 methylomes is a massive undertaking, and this manuscript provides a highly valuable resource to the aging research community.
I commend the authors for their diligent efforts in addressing the concerns raised by all reviewers during the first round. The revisions have significantly elevated the statistical rigor, clarity, and interpretability of the study.
Strengths of the Revisions:
Cell-Type Deconvolution: The addition of cell-type composition adjustments using EpiScore and EpiDISH for the applicable tissues is a major and necessary improvement. Demonstrating that the t-statistics of age-associated CpGs remain highly correlated (to) before and after adjustment (Supplementary Figure 2) provides strong confidence that the primary signals represent true intrinsic epigenetic remodeling rather than mere compositional shifts.
Power Calculations: The newly added empirical power calculations (Supplementary Table 4) perfectly address the concerns regarding the lack of signals in tissues like the prostate, stomach, and pancreas. Proving that these null results are driven by low detectability/sample size rather than a biologically static epigenome adds crucial context and prevents misinterpretation of the atlas.
Toned-down Causality: The authors appropriately adjusted their terminology, replacing terms like "driver genes" with "module-influential genes" or "network-central genes." This linguistic shift accurately aligns with the correlational and in silico nature of the WGCNA and perturbation analyses.
Figure Formatting: The aesthetic and structural overhauls to the figures (particularly the shift to stacked bar charts in Figure 2 and standardizing the multi-panel layouts) have vastly improved the reader's ability to make cross-tissue comparisons.

Minor points:
1.Terminology Consistency: While the authors successfully removed "driver genes," the term "global disruptor" is still utilized in the main text (e.g., Line 419: "a limited set of 'global disruptor' genes"). While acceptable within the context of the in silico robustness simulations, I recommend adding a brief, one-sentence inline clarification when the term is first introduced in the main text to remind readers that this refers strictly to network topology and simulated mathematical disruption, not a biologically validated causal disruption.

2.Figure Presentation and Legends: Please further optimize the aesthetic layout of the figures and refine the accompanying legends. Currently, the captions for the Supplementary Figures appear somewhat sparse. Expanding these legends to be more comprehensive, consistent, and standardized will greatly assist readers in fully grasping the complex visual information without needing to constantly cross-reference the main text.
3.Supplementary Callouts: please check the supplementary callouts in the text. For instance, on Line 421, 425 and 434, the text references "(Supplementary Table 7)" three times in close succession regarding module disruption; ensure the referencing flows naturally.
Overall, this is an excellent, rigorous, and highly comprehensive study. The authors have fully satisfied the requirements of the peer review process.

(Remarks on code availability)

Reviewer #2

(Remarks to the Author)
>>> Reviewer #2 Responses

Jacques et al. have submitted a Resource manuscript to Nature Aging interrogating ageing-
related DNA methylation (DNAm) changes in ~15k DNAm array samples across 17 tissues. The tissue sets comprising this meta-analysis range in size from ~7k samples for brain- derived to 73 from the Pancreas. However, it importantly excluded the much larger blood-derived datasets available in order not to be heavily biased by that well-analysed tissue type.
The authors analysed this dataset for DMPs, VMPs, and Shannon entropy. Interestingly, the authors identified through network analysis and shared signatures, genes and gene families with cross-tissue driver effects, such as Protocadherin gamma, HOX, and Histone Deacetylase 4. These genes and pathways highlight candidates for further follow-up work. Additionally, they state they provide these data as a browsable resource for the community. This work is novel and of broad interest to a wide audience - as analysis of the ageing DNA methylome is a very active area of research, and most previous work has been biased towards blood-derived results. However, some of the interpretations made in the manuscript could be more nuanced. My comments for the authors to consider are listed below

Major1. The R shiny application link was not working at my time of access.

Response: We apologise for the inconvenience caused by the temporary inaccessibility of the R Shiny application at the time of access. To accommodate the increasing volume of data and improve stability and performance, the application has now been migrated to a new server. The updated and fully functional link is:
https://bioinformatics.erc.monash.edu/apps/human-aging-atlas/
We have updated the manuscript accordingly to reflect this change.

>>> Good this is now accessible. All data from this analysis presented - however, DMP blood tab gave error message at the time of access.

2. The authors acknowledge the major issue of cell type heterogeneity within these tissues –
however, this needs to be considered regarding many of the statements about tissue-specific differences. For example, it may be that tissues showing strong intermediate methylation signals are due to a predominant mixture of an unmethylated cell-type/s and a methylated cell type/s. These are observed to start at an intermediate level and that rises as the unmethylated cell/s hypermethylated with age. This should be considered along with statements about ‘progressive chromatin condensation and regional silencing as a potential aging mechanism’. Also, regarding the statement that ‘skeletal muscle and lung tissues exhibited 61% and 50%, respectively, of DMPs in the intermediate methylation state’.

Response: We thank the reviewer for this thoughtful and important comment. To address this, we have now applied EpiScore1 to estimate and adjust for cell-type composition in tissues with high-confidence reference panels. This approach helps to mitigate potential confounding arising from mixtures of methylated and unmethylated cell populations within bulk samples. Additional results can be found on: Page 5, Lines 177-183, Page 7, Lines 241-244, Supplementary Table 3, Supplementary Figure 2.
In the revised Discussion, we now explicitly acknowledge that some of the observed “intermediate” methylation states may reflect differences in cellular composition rather than purely intrinsic epigenetic intermediacy. We have also qualified our interpretation of “progressive chromatin condensation and regional silencing” to note that this trend may partially arise from age-related shifts in cell-type proportions (page 11, lines 452-456 and 463-464). Corresponding text was added to the Discussion and Limitations sections (page 15, line 620-622) to clarify this point and ensure balanced interpretation.
Overall, while we recognise that bulk data cannot fully disentangle cell-type–specific dynamics, the inclusion of EpiScore-based adjustments shows that age-related shifts in cell proportions affect different tissues to varying degrees. However, most age-associated methylation patterns largely reflect genuine biological aging effects rather than compositional artefacts.

>>> Good that the authors now acknowledge cell type heterogeneity as a contributor to the observed intermediate methylation.

3. Regarding the Methodology – could the authors comment on whether they considered
using unsupervised reference-free methods to attempt to capture cell-type effects (SVA etc)?

Response: We thank the reviewer for this suggestion. We agree that unsupervised, reference-free methods such as SVA can in principle help account for unobserved sources of variation, including cell-type effects. Following the reviewers' suggestions, we relied on a combination of established pre-processing and normalization steps implemented by EPISCORE algorithm1 for tissues with high-confidence reference panels to address potential cellular heterogeneity directly. Reference-based methods generally work better than reference-free methods, a point made in here3. We have clarified this in the Methods section (page 16 – lines 611-629).

>>> Good to explore this

4. The authors used 27k, 450k, and EPIC DNAm – all DNAm arrays are biased compared to the genomic distribution of CpGs. However, the arrays have variation in this bias, such as differing enrichment of CpG island regions – with the 27k array being predominantly CpGs in dense CpG regions. It may have been factored in, but it was not clear to me whether the differing proportions of array type in the different tissues were accounted for - i.e., more contributed to by 27k (e.g., Prostate is one third 27k). If not, this may have influenced the tissue summary results – e.g. greater proportion of CpG in CpG islands. Did CpG island array bias (27k > 450k > EPIC) contribute to any of the signals driving 'broad upregulation of pathways tied to transcriptional regulation'?

Response: We thank the reviewer for this thoughtful and important point regarding array- specific CpG coverage bias, particularly the differential enrichment of CpG island regions across the 27k, 450k, and EPIC platforms. We agree that these arrays differ in genomic coverage, with the 27k array being heavily biased toward CpG-dense regions, and that unequal representation across tissues could, in principle, influence tissue-level summaries if not appropriately handled.
In our analytical framework, this potential bias is mitigated at multiple levels. First, all association analyses were performed within each dataset independently, such that age effects were estimated relative to the probe content of that specific array. This ensures that comparisons are never made directly at the probe level across platforms. Second, tissue-level integration was performed using summary statistic–based meta-analysis, which combines effect sizes across independent studies and arrays. This approach reduces the influence of platform-specific probe distributions, as consistent directionality and magnitude of association across studies are prioritised, while array-specific effects are diluted rather than amplified. Importantly, technical differences between methylation arrays predominantly affect CpG representation and effect size magnitude, but are less likely to induce systematic, directionally consistent age associations across independent datasets. As such, the pathway-level signals identified, particularly those emerging from gene-level aggregation and directionality-aware analyses (e.g., mitch), are unlikely to be driven solely by CpG island overrepresentation in specific arrays. Moreover, the observation of concordant pathway-level
shifts across tissues profiled using different array compositions further argues against array bias as the primary driver of these findings.
We have now clarified this rationale in the Methods (Page 16, lines 664 to 679) to improve transparency.

>>> Excellent that the authors have now detailed their consideration of the differing array platforms in the analytical processing.

5. The authors stated that '65% of DMPs in the young subjects were from low methylation regions'. They should also explore this further by defining the CpG% density (as well as island/shore classification) of these defined low methylation regions. Thus, they could add further details to the statement that in 'most tissues, age-DMPs were primarily found in regions with low methylation (<25%)'.

Response: We agree that the genomic context of low-methylation regions provides important insights into their regulatory relevance. In the revised manuscript, we have now annotated all DMPs according to CpG island context (island, shore, shelf, open sea) and local CpG density. This analysis revealed that the majority of low-methylation DMPs (<25% in young individuals) were located in CpG-sparse (open sea) and shore regions, consistent with prior evidence that age-associated hypermethylation frequently occurs in regulatory elements outside CpG islands. We have added these results to the Results section and included a summary figure (Supplementary Fig. 3, Page 5 lines 180-183) showing the distribution of age-DMPs across CpG density and island categories.
These additional annotations reinforce our conclusion that age-related DMPs predominantly occur in low-methylation, CpG-sparse regions, likely reflecting enhancer- or intergenic-level remodelling during aging.

>>> Excellent to provide this illuminating additional information on genomic context and CpG density.

6. The VMP analysis showed minimal consistent findings – was there any evidence that these CpGs are more confounded genetically – i.e. were they enriched for being CpGs under mQTL effects? [1].

Response: While it is well established that CpGs under strong mQTL control can exhibit higher inter-individual variability, our VMP analysis specifically tested for age-associated changes in variance rather than absolute variability. Genetic effects on DNA methylation are generally stable across the lifespan and therefore would not be expected to generate age-dependent increases or decreases in variance unless interacting with age. Importantly, all CpG sites were included in the analysis without prior filtering for mQTL status, ensuring that the statistical framework did not preferentially select genetically driven loci. Thus, any observed VMPs reflect loci in which methylation variance changes with age, independent of baseline genetic regulation. While enrichment analysis for known mQTLs could provide additional annotation, such analysis would not alter the primary interpretation that these loci demonstrate age-associated variance dynamics.
We have clarified this point in the Discussion (Page 12, Lines: 481-486) to distinguish genetically driven methylation variability from age-associated variance effects.

>>> Good that the authors have included further Discussion re this.

7. The authors acknowledge the likely reduced ability to detect DMPs in tissues with limited samples, but did they perform specific power calculations for each tissue [2] (also taking into consideration the array type contributions to each tissue set) that could then be used to inform on the results observed?

Results: We thank the reviewer for this important suggestion. We have now performed tissue- specific detectability analyses using the meta-analysis summary statistics (pooled effect sizes and standard errors from METAL). For each tissue, we derived the empirical P-value threshold corresponding to FDR ≤ 0.005 and converted this to an equivalent Z-score threshold. Using these tissue-specific thresholds and observed variance structure, we estimated the power to detect representative effect sizes (median and 75th percentile absolute β values). This analysis demonstrated that tissues with small sample sizes (e.g., heart, pancreas, prostate, stomach) had extremely low power to detect median observed effect sizes at FDR ≤0.005, explaining the limited number of significant associations in these tissues. In contrast, large tissues such as brain (N = 7,451), showed substantial power to detect upper-quartile effects (>70%), consistent with the high discovery burden observed.
Interestingly, lung and liver exhibited relatively high detectability for stronger effects (61% and 49%, respectively) despite moderate sample sizes, reflecting comparatively larger effect sizes and favorable variance structure. Conversely, muscle (N = 1,568), although well powered by sample size, showed generally smaller effect sizes, resulting in more modest detectability (~15% for upper-quartile effects). These findings indicate that tissue-specific discovery rates are driven by both sample size and the underlying distribution of effect sizes, rather than sample size alone.
All individual studies were corrected for inflation using the bacon method prior to meta-analysis, ensuring that the pooled test statistics and standard errors used for detectability estimation were not inflated. Because the meta-analysis standard errors inherently reflect study-level variance (including array type, batch effects, and sample size), array contributions are accounted for within the pooled variance structure. These results have been added to the manuscript (page 6 lines 200-210, Supplementary Table 4).

>>> Good to acknowledge this power differential between tissues transparently now.

8. Blood infiltration/contamination of tissue samples is another issue that could be assessed by cellular deconvolution.

Response: We thank the reviewer for this important point. Cellular deconvolution was performed using EPISCORE48 for all tissues except buccal, where EpiDISH49 was used EpiDISH, and the estimated cell-type proportions, including immune-related fractions, were incorporated as covariates in the regression models. Therefore, potential blood infiltration or immune cell contamination was explicitly accounted for in the adjusted analyses. As shown by the comparison between adjusted and unadjusted models, a subset of age-associated signals was attenuated or increased, depending on the tissue, following correction, indicating that part of the bulk signal reflects compositional effects. However, a substantial number of DMPs remained significant after adjustment, supporting the presence of cell-intrinsic age- associated methylation changes beyond blood infiltration effects. We have clarified this in the Methods (Page 17, lines 710-726) and Results sections (Page 6, lines 200-210 & Page 7, lines 242-245).

>>> Good to include this point now.

(Remarks on code availability)

Reviewer #3

(Remarks to the Author)
The authors have submitted an improved manuscript and addressed many of our suggestions. However they continue to overstate the conclusions that can be made as a result of their study, in particular the implications of their findings on the relevance of stochastic drift to epigenetic aging.

- The authors did not make any attempt to address our point #3, barely changing the original figure. They should re-attempt to address our original comment as a figure that allows easy comparison of entropic change across tissues is of utmost importance for conveying the results. The change the authors made to Figure 2 was very nice – something similar here would be helpful. Original comment: " Figure 4. A figure which shows the effect size of entropy gain/loss in each tissue should be added to allow comparison across tissues. This could be a barplot where for each tissue there are 4 bars the height of each corresponds to the slope of entropy gain/loss for that class of CpG sites in that tissue. The statistical of this slope should be reported along with the effect size (in the figure)."

- The authors have inadequately addressed our previous comment (#6) regarding entropic/stochastic epigenetic drift during aging. They write "The predominance of age-associated hypermethylation across multiple tissues challenges the prevailing notion that aging primarily reflects stochastic drift toward intermediate methylation states." This is not an accurate conclusion from the data. As they nicely present, the majority of tissues begin in a state of hypomethylation (Figure 2). Thus, if every CpG merely drifted towards an intermediate methylation state, age-associated hypermethylation is exactly what would be expected. They also argue that "directional methylation shifts across tissues, enrichment within defined biological pathways,

and tissue-dependent entropy dynamics based on Shannon entropy analysis" rebut the idea of stochastic drift. This is false. Each of these findings are plausible under pure stochstic drift assuming each tissue & biological pathway has a different starting methylation pattern. To amend this, the authors should significantly revise all portions of the manuscript which address the idea of stochasticity in epigenetic aging. If they want to contribute to this important area of research they should conduct a more nuanced analysis. Reflection upon these studies may assist the authors in this endeavor: Myer et al. 2025, Tarkhov et al. 2024, Tong et al. 2024.

- The authors did not fully address our comment #7. The sentence we quote there – which wildly overstates their findings – is still present in the abstract. The authors should choose a more causally neutral term than "disruptor" there and throughout the manuscript.

- While some figures have been improved, figures 4-6 have highly illegible text (We tested this in 2 pdf viewers). This may be amended by increased text size, reduced figure complexity, or increased resolution.

(Remarks on code availability)

Open Access This Peer Review File is licensed under a Creative Commons Attribution 4.0 International License, which permits use, sharing, adaptation, distribution and reproduction in any medium or format, as long as you give appropriate credit to the original author(s) and the source, provide a link to the Creative Commons license, and indicate if changes were made.
In cases where reviewers are anonymous, credit should be given to 'Anonymous Referee' and the source.
The images or other third party material in this Peer Review File are included in the article’s Creative Commons license, unless indicated otherwise in a credit line to the material. If material is not included in the article’s Creative Commons license and your intended use is not permitted by statutory regulation or exceeds the permitted use, you will need to obtain permission directly from the copyright holder.
To view a copy of this license, visit https://creativecommons.org/licenses/by/4.0/

Dear Editor,

We thank the editorial team and reviewers for their time and effort in evaluating our manuscript, **DNA Methylation Ageing Atlas Across 17 Human Tissues**, *Nature Aging*, and for emphasising the significance of the topic. We have addressed all reviewer comments and provided detailed responses. Notably, we extended our analysis to include WGCNA across all tissues and cell type deconvolution, as recommended by the reviewers. Please see below for detailed responses.

Reviewer #1 :

This manuscript presents a comprehensive DNA methylation aging atlas across 17 human tissues, analyzing over 15,000 methylomes through multidimensional approaches including DMPs, VMPs, and Shannon entropy. The study identifies both tissue-specific and conserved epigenetic aging signatures, with network analyses revealing key driver genes and pathways. The authors provide a valuable open-access resource for investigating molecular aging mechanisms. This study represents a significant contribution to the epigenetics of aging field. I have following suggestions to improve the paper.

1. The substantial disparity in sample sizes across tissues (e.g., limited samples for prostate, rectum, and stomach) raises concerns about detection power and comparative reliability. The authors should discuss how this imbalance affects cross-tissue comparisons and whether statistical corrections were adequately applied.

Response: We thank the reviewer for this important point. We agree that smaller sample sizes in certain tissues (e.g., prostate, rectum, and stomach) may reduce statistical power and limit the detection of significant CpG-level changes across some analytical layers. We now explicitly acknowledge this limitation in the manuscript (page 11, lines 440–448).

Importantly, to mitigate the impact of sample size imbalance on cross-tissue comparisons, we employed a directionality-based pathway analysis using mitch (https://pmc.ncbi.nlm.nih.gov/articles/PMC7325150/; see page 19, lines 793–805). This approach does not rely on CpG-level p-values, but instead integrates the direction and consistency of change across groups of CpGs mapped to genes and pathways. As a result, coordinated shifts in methylation can still be captured even in tissues where individual CpGs may not reach statistical significance due to limited power.

This strategy allowed us to retain under-represented tissues in cross-tissue analyses while reducing bias introduced by unequal sample sizes, and we now clarify this rationale in the manuscript.

2. The use of bulk tissue data without proper cell-type composition adjustment may confound observed methylation patterns. Age-related changes in cell population proportions could mimic epigenetic aging signals. Cellular deconvolution analysis or detailed discussion of this limitation is warranted.

Response: We agree that cellular heterogeneity can influence DNA methylation patterns and potentially confound age-related associations. To address this, in the revised analysis we have applied EPISCORE[1] for all tissues except buccal, where EpiDISH[2] was used to

estimate and adjust for cell-type composition in tissues with high-confidence reference panels. This approach enables partial deconvolution of bulk methylation data and helps mitigate potential biases arising from age-dependent shifts in cell-type proportions (Page 5, Lines 177-184, Page 7, Lines 242-245, Supplementary Table 3, Supplementary Figures 2).

To evaluate the impact of this adjustment, we compared age-association statistics derived from models before and after cell-type adjustment across all tissues. As shown in Supplementary Figure 2, the t-statistics of age-associated CpGs were highly correlated between unadjusted and adjusted analyses, with correlation coefficients ranging from r = 0.81 to r = 0.99 across tissues. This strong concordance indicates that the majority of identified age-associated methylation signals are robust to cell-type composition correction.

In addition, we examined the overlap of variably methylated positions (VMPs) identified before and after adjustment. Across tissues, there was substantial overlap between the two analyses, further supporting the stability of the detected age-related methylation variability patterns. These results suggest that while cellular composition may contribute modestly to some signals, the dominant age-associated methylation patterns reported in this study are largely independent of cell-type proportion changes.

Together, these additional analyses suggest that the observed age-related methylation signatures reflect true epigenetic alterations rather than artifacts of tissue cellular heterogeneity.

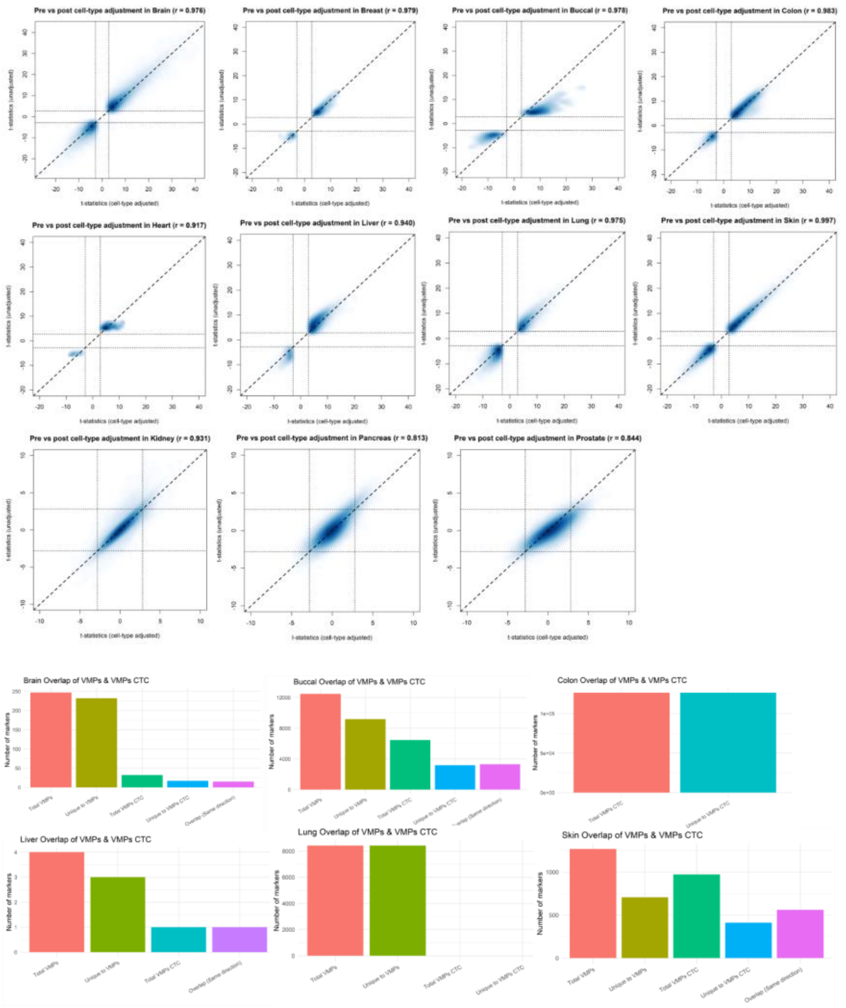

Supplementary Figure 2 Correlation plot between DMPs identifies with cell-type correction (x-axis) and without cell-type correction (y-axis) and number of VMPs identified before and after cell-type correction

3. The exclusive reliance on cross-sectional data prevents distinction between true aging effects and cohort differences. Longitudinal validations of identified epigenetic signatures are critical and would significantly strengthen the findings.

Response: We agree with the reviewer that longitudinal data are essential to definitively disentangle true aging effects from cohort-related differences, and that longitudinal validation would further strengthen the findings. However, the present study is necessarily constrained by the availability of open-access human epigenomic datasets, for which longitudinal sampling across multiple tissues is currently extremely limited. Repeated sampling of most solid tissues in humans is not feasible for ethical and practical reasons, making large-scale longitudinal designs rare in this field.

Within these constraints, we have been careful to frame our results as cross-sectional aging associations rather than definitive within-individual aging trajectories. Importantly, our analyses leverage consistency of methylation patterns across tissues and independent cohorts to reduce the likelihood that observed signals reflect cohort-specific effects alone. Nonetheless, we now explicitly highlight this limitation and opportunity in the Discussion (page 14, lines 559-583).

4. While bioinformatic analyses are comprehensive, experimental validation of key discoveries (particularly the proposed central role of PCDHG gene family) is missing. Integration with expression data or functional assays would enhance mechanistic insights.

Response: We thank the reviewer for this important suggestion. The primary aim of this study, however, is to present a comprehensive, cross-tissue epigenomic resource and analytical framework to identify robust, reproducible aging-associated signatures across human tissues.

Given the scale and scope of the resource generated, integrating functional assays within the same manuscript would substantially expand its focus and is beyond the intended scope of the present study. Importantly, we view the identification of PCDHG genes as high-confidence candidate regulators rather than definitive mechanistic endpoints. Consistent with the reviewer's suggestion, follow-up studies are currently underway, including CRISPR-based perturbation experiments, chromatin accessibility profiling, and cell-based modeling, to directly test the functional consequences of epigenetic regulation at these loci. These efforts will be presented in a dedicated follow-up study focused on mechanistic validation.

5. The combination of 131 datasets from different platforms and batches, despite statistical corrections, requires more rigorous demonstration of data harmonization efficacy and consistency across technical variations.

Response: To minimise technical heterogeneity, each dataset was pre-processed independently using an identical and standardised pipeline, rather than applying joint normalisation across studies. This approach was deliberately chosen to avoid introducing cross-study pre-processing artefacts and to ensure that platform- and study-specific technical characteristics were handled locally and consistently (page 15, lines 631-646).

Importantly, all association analyses were first performed within each individual dataset, with models explicitly correcting for cohort-specific confounders and technical covariates where available. This study-level modelling strategy reduces heterogeneity arising from differences in study design, platform, and batch structure before any cross-study integration (See Supplementary Table 1 & page 15, line 648 to 657).

Downstream integration was performed at the level of summary statistics using meta-analysis, which further mitigates residual technical variability by combining effect sizes across multiple independent studies. In the context of DNA methylation data, technical variation across platforms and batches is typically small, while absolute effect magnitudes may vary, the direction of association is generally preserved. Furthermore, we have not merged datasets, so batch effects would be an even smaller issue. Meta-analysis therefore provides a robust framework to prioritise consistent, directionally concordant signals while reducing the likelihood of technical artefact–driven findings (Page 16, lines 648 to 663).

6. The multilayer analytical approach (DMPs, VMPs, entropy, WGCNA, mitch) creates substantial multiple testing burden. Clearer description of overall false discovery rate control strategies is needed.

Response: We carefully considered false discovery control at each stage of the analysis and adopted a hierarchical and conservative strategy rather than applying a single global correction across fundamentally distinct analytical layers.

Specifically, false discovery rate (FDR) correction was applied independently within each analysis layer (DMPs, VMPs, entropy, WGCNA modules, and pathway enrichment), using a stringent threshold of FDR < 0.005 throughout. This threshold is substantially more conservative than commonly used cut-offs in epigenome-wide studies and was chosen to limit false positives given the scale of testing performed.

Importantly, downstream integration focused on signals that were reproducible across tissues, datasets, or analytical layers, providing an additional level of validation beyond formal multiple testing correction. We note that applying a single joint correction across all layers would be statistically inappropriate, as each layer tests distinct hypotheses with different dependency structures.

We have now clarified this overall false discovery control strategy and its rationale in the Methods to improve transparency and reproducibility (Page 14, lines 600 to 605).

7. The potential implications for biomarkers and therapeutic targets require more substantive discussion regarding practical applications in age-related disease prediction and intervention strategies.

Response: We have expanded the Discussion to more explicitly address the translational implications of our findings, including how cross-tissue and tissue-specific epigenetic signatures may be leveraged for biomarker development and how network-level vulnerabilities identify candidate pathways for therapeutic intervention (Page 14, lines 551 to 587).

Minor points

1. Key terms like "fragile modules" and "resilient modules" require more precise definitions, especially when discussing their functional significance.

Response: We thank the reviewer for this suggestion and agree that clearer definitions of “fragile” and “resilient” modules are warranted. In this study, we define fragile modules as co-methylation gene networks whose structure and aging-associated signatures are strongly disrupted by simulated perturbation of individual or small sets of genes, indicating high sensitivity to epigenetic changes. In contrast, resilient modules are defined as networks that maintain their overall structure and aging-associated signal despite comparable perturbations, reflecting greater robustness to epigenetic variation.

We have now added explicit definitions of these terms and clarified their functional interpretation in the Methods (page 21, lines 844 to 848).

2. Some conclusions appear overly definitive for correlative findings (e.g., definitive statements about PCDHG gene family roles). More cautious language reflecting the observational nature of the study is appropriate.

Response: We have revised the text to adopt more cautious language when discussing the PCDHG gene family, framing these genes as high-confidence, cross-tissue candidates prioritised by integrative epigenomic analyses rather than definitive drivers of aging. We now explicitly emphasize that the associations reported here are correlative and that functional validation is required to establish causality.

3. While methods are generally detailed, certain parameter selections (e.g., WGCNA soft-thresholding criteria) need clearer justification.

Response: For WGCNA, the soft-thresholding power was selected using the standard scale-free topology criterion, choosing the lowest power at which the scale-free topology fit index (R^2) exceeded 0.8 while maintaining adequate mean connectivity. This data-driven approach is widely recommended in the WGCNA framework to balance network robustness and biological interpretability.

To ensure that results were not unduly sensitive to this choice, we verified that module structure and age associations were stable across a narrow range of neighbouring powers. We have now clarified this rationale and added the relevant details to the Methods.

Reviewer #1 (Remarks on code availability):

It is adequate

Reviewer #2 :

Jacques et al. have submitted a Resource manuscript to Nature Aging interrogating ageing-related DNA methylation (DNAm) changes in ~15k DNAm array samples across 17 tissues. The tissue sets comprising this meta-analysis range in size from ~7k samples for brain-derived to 73 from the Pancreas. However, it importantly excluded the much larger blood-derived datasets available in order not to be heavily biased by that well-analysed tissue type.

The authors analysed this dataset for DMPs, VMPs, and Shannon entropy. Interestingly, the authors identified through network analysis and shared signatures, genes and gene families with cross-tissue driver effects, such as Protocadherin gamma, HOX, and Histone Deacetylase 4. These genes and pathways highlight candidates for further follow-up work. Additionally, they state they provide these data as a browsable resource for the community. This work is novel and of broad interest to a wide audience - as analysis of the ageing DNA methylome is a very active area of research, and most previous work has been biased towards blood-derived results. However, some of the interpretations made in the manuscript could be more nuanced. My comments for the authors to consider are listed below

Major

1. The R shiny application link was not working at my time of access.

Response: We apologise for the inconvenience caused by the temporary inaccessibility of the R Shiny application at the time of access. To accommodate the increasing volume of data and improve stability and performance, the application has now been migrated to a new server. The updated and fully functional link is:

https://bioinformatics.erc.monash.edu/apps/human-aging-atlas/

We have updated the manuscript accordingly to reflect this change.

2. The authors acknowledge the major issue of cell type heterogeneity within these tissues – however, this needs to be considered regarding many of the statements about tissue-specific differences. For example, it may be that tissues showing strong intermediate methylation signals are due to a predominant mixture of an unmethylated cell-type/s and a methylated cell type/s. These are observed to start at an intermediate level and that rises as the unmethylated cell/s hypermethylated with age. This should be considered along with statements about 'progressive chromatin condensation and regional silencing as a potential aging mechanism'. Also, regarding the statement that 'skeletal muscle and lung tissues exhibited 61% and 50%, respectively, of DMPs in the intermediate methylation state'.

Response: We thank the reviewer for this thoughtful and important comment. To address this, we have now applied EpiScore[1] to estimate and adjust for cell-type composition in tissues with high-confidence reference panels. This approach helps to mitigate potential confounding arising from mixtures of methylated and unmethylated cell populations within bulk samples. Additional results can be found on: Page 5, Lines 177-183, Page 7, Lines 241-244, Supplementary Table 3, Supplementary Figure 2.

In the revised Discussion, we now explicitly acknowledge that some of the observed "intermediate" methylation states may reflect differences in cellular composition rather than purely intrinsic epigenetic intermediacy. We have also qualified our interpretation of "progressive chromatin condensation and regional silencing" to note that this trend may partially arise from age-related shifts in cell-type proportions (page 11, lines 452-456 and 463-464). Corresponding text was added to the Discussion and Limitations sections (page 15, line 620-622) to clarify this point and ensure balanced interpretation.

Overall, while we recognise that bulk data cannot fully disentangle cell-type–specific dynamics, the inclusion of EpiScore-based adjustments shows that age-related shifts in cell proportions affect different tissues to varying degrees. However, most age-associated methylation patterns largely reflect genuine biological aging effects rather than compositional artefacts.

3. Regarding the Methodology – could the authors comment on whether they considered using unsupervised reference-free methods to attempt to capture cell-type effects (SVA etc)?

Response: We thank the reviewer for this suggestion. We agree that unsupervised, reference-free methods such as SVA can in principle help account for unobserved sources of variation, including cell-type effects. Following the reviewers' suggestions, we relied on a combination of established pre-processing and normalization steps implemented by EPISCORE algorithm[1] for tissues with high-confidence reference panels to address potential cellular heterogeneity directly. Reference-based methods generally work better than reference-free methods, a point made in here[3]. We have clarified this in the Methods section

(page 16 – lines 611-629).

4. The authors used 27k, 450k, and EPIC DNAm – all DNAm arrays are biased compared to the genomic distribution of CpGs. However, the arrays have variation in this bias, such as differing enrichment of CpG island regions – with the 27k array being predominantly CpGs in dense CpG regions. It may have been factored in, but it was not clear to me whether the differing proportions of array type in the different tissues were accounted for - i.e., more contributed to by 27k (e.g., Prostate is one third 27k). If not, this may have influenced the tissue summary results – e.g. greater proportion of CpG in CpG islands. Did CpG island array bias (27k > 450k > EPIC) contribute to any of the signals driving 'broad upregulation of pathways tied to transcriptional regulation'?

Response: We thank the reviewer for this thoughtful and important point regarding array-specific CpG coverage bias, particularly the differential enrichment of CpG island regions across the 27k, 450k, and EPIC platforms. We agree that these arrays differ in genomic coverage, with the 27k array being heavily biased toward CpG-dense regions, and that unequal representation across tissues could, in principle, influence tissue-level summaries if not appropriately handled.

In our analytical framework, this potential bias is mitigated at multiple levels. First, all association analyses were performed within each dataset independently, such that age effects were estimated relative to the probe content of that specific array. This ensures that comparisons are never made directly at the probe level across platforms. Second, tissue-level integration was performed using summary statistic–based meta-analysis, which combines effect sizes across independent studies and arrays. This approach reduces the influence of platform-specific probe distributions, as consistent directionality and magnitude of association across studies are prioritised, while array-specific effects are diluted rather than amplified.

Importantly, technical differences between methylation arrays predominantly affect CpG representation and effect size magnitude, but are less likely to induce systematic, directionally consistent age associations across independent datasets. As such, the pathway-level signals identified, particularly those emerging from gene-level aggregation and directionality-aware analyses (e.g., mitch), are unlikely to be driven solely by CpG island overrepresentation in specific arrays. Moreover, the observation of concordant pathway-level shifts across tissues profiled using different array compositions further argues against array bias as the primary driver of these findings.

We have now clarified this rationale in the Methods (Page 16, lines 664 to 679) to improve transparency.

5. The authors stated that '65% of DMPs in the young subjects were from low methylation regions'. They should also explore this further by defining the CpG% density (as well as island/shore classification) of these defined low methylation regions. Thus, they could add further details to the statement that in 'most tissues, age-DMPs were primarily found in regions with low methylation (<25%)'.

Response: We agree that the genomic context of low-methylation regions provides important insights into their regulatory relevance. In the revised manuscript, we have now annotated all DMPs according to CpG island context (island, shore, shelf, open sea) and local CpG density. This analysis revealed that the majority of low-methylation DMPs (<25% in young individuals) were located in CpG-sparse (open sea) and shore regions, consistent with prior evidence that age-associated hypermethylation frequently occurs in regulatory elements

outside CpG islands. We have added these results to the Results section and included a summary figure (Supplementary Fig. 3, Page 5 lines 180-183) showing the distribution of age-DMPs across CpG density and island categories.

These additional annotations reinforce our conclusion that age-related DMPs predominantly occur in low-methylation, CpG-sparse regions, likely reflecting enhancer- or intergenic-level remodelling during aging.

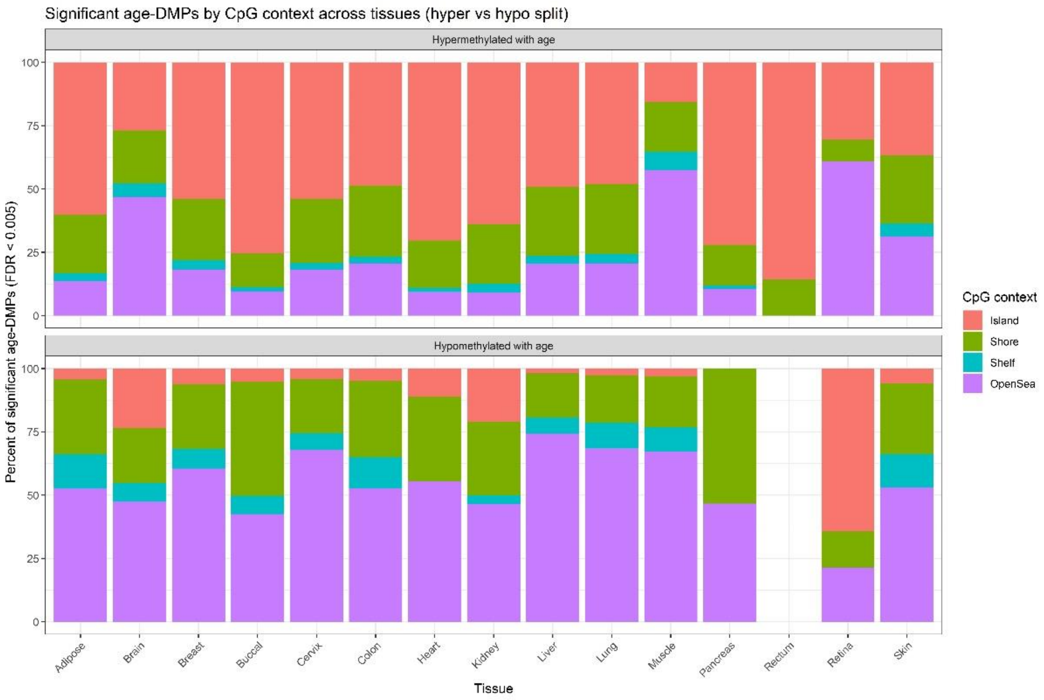

6. The VMP analysis showed minimal consistent findings – was there any evidence that these CpGs are more confounded genetically – i.e. were they enriched for being CpGs under mQTL effects? [1].

Response: While it is well established that CpGs under strong mQTL control can exhibit higher inter-individual variability, our VMP analysis specifically tested for age-associated changes in variance rather than absolute variability. Genetic effects on DNA methylation are generally stable across the lifespan and therefore would not be expected to generate age-dependent increases or decreases in variance unless interacting with age.

Importantly, all CpG sites were included in the analysis without prior filtering for mQTL status, ensuring that the statistical framework did not preferentially select genetically driven loci. Thus, any observed VMPs reflect loci in which methylation variance changes with age, independent of baseline genetic regulation. While enrichment analysis for known mQTLs could provide additional annotation, such analysis would not alter the primary interpretation that these loci demonstrate age-associated variance dynamics.

We have clarified this point in the Discussion (Page 12, Lines: 481-486) to distinguish genetically driven methylation variability from age-associated variance effects.

7. The authors acknowledge the likely reduced ability to detect DMPs in tissues with limited

samples, but did they perform specific power calculations for each tissue [2] (also taking into consideration the array type contributions to each tissue set) that could then be used to inform on the results observed?

Results: We thank the reviewer for this important suggestion. We have now performed tissue-specific detectability analyses using the meta-analysis summary statistics (pooled effect sizes and standard errors from METAL). For each tissue, we derived the empirical P-value threshold corresponding to FDR ≤ 0.005 and converted this to an equivalent Z-score threshold. Using these tissue-specific thresholds and observed variance structure, we estimated the power to detect representative effect sizes (median and 75th percentile absolute β values).

This analysis demonstrated that tissues with small sample sizes (e.g., heart, pancreas, prostate, stomach) had extremely low power to detect median observed effect sizes at FDR ≤ 0.005, explaining the limited number of significant associations in these tissues. In contrast, large tissues such as brain (N = 7,451), showed substantial power to detect upper-quartile effects (>70%), consistent with the high discovery burden observed.

Interestingly, lung and liver exhibited relatively high detectability for stronger effects (61% and 49%, respectively) despite moderate sample sizes, reflecting comparatively larger effect sizes and favorable variance structure. Conversely, muscle (N = 1,568), although well-powered by sample size, showed generally smaller effect sizes, resulting in more modest detectability (~15% for upper-quartile effects). These findings indicate that tissue-specific discovery rates are driven by both sample size and the underlying distribution of effect sizes, rather than sample size alone.

All individual studies were corrected for inflation using the bacon method prior to meta-analysis, ensuring that the pooled test statistics and standard errors used for detectability estimation were not inflated. Because the meta-analysis standard errors inherently reflect study-level variance (including array type, batch effects, and sample size), array contributions are accounted for within the pooled variance structure.

These results have been added to the manuscript (page 6 lines 200-210, Supplementary Table 4).

8. Blood infiltration/contamination of tissue samples is another issue that could be assessed by cellular deconvolution.

Response: We thank the reviewer for this important point. Cellular deconvolution was performed using EPISCORE[48] for all tissues except buccal, where EpiDISH[49] was used EpiDISH, and the estimated cell-type proportions, including immune-related fractions, were incorporated as covariates in the regression models. Therefore, potential blood infiltration or immune cell contamination was explicitly accounted for in the adjusted analyses. As shown by the comparison between adjusted and unadjusted models, a subset of age-associated signals was attenuated or increased, depending on the tissue, following correction, indicating that part of the bulk signal reflects compositional effects. However, a substantial number of DMPs remained significant after adjustment, supporting the presence of cell-intrinsic age-associated methylation changes beyond blood infiltration effects. We have clarified this in the Methods (Page 17, lines 710-726) and Results sections (Page 6, lines 200-210 & Page 7, lines 242-245).

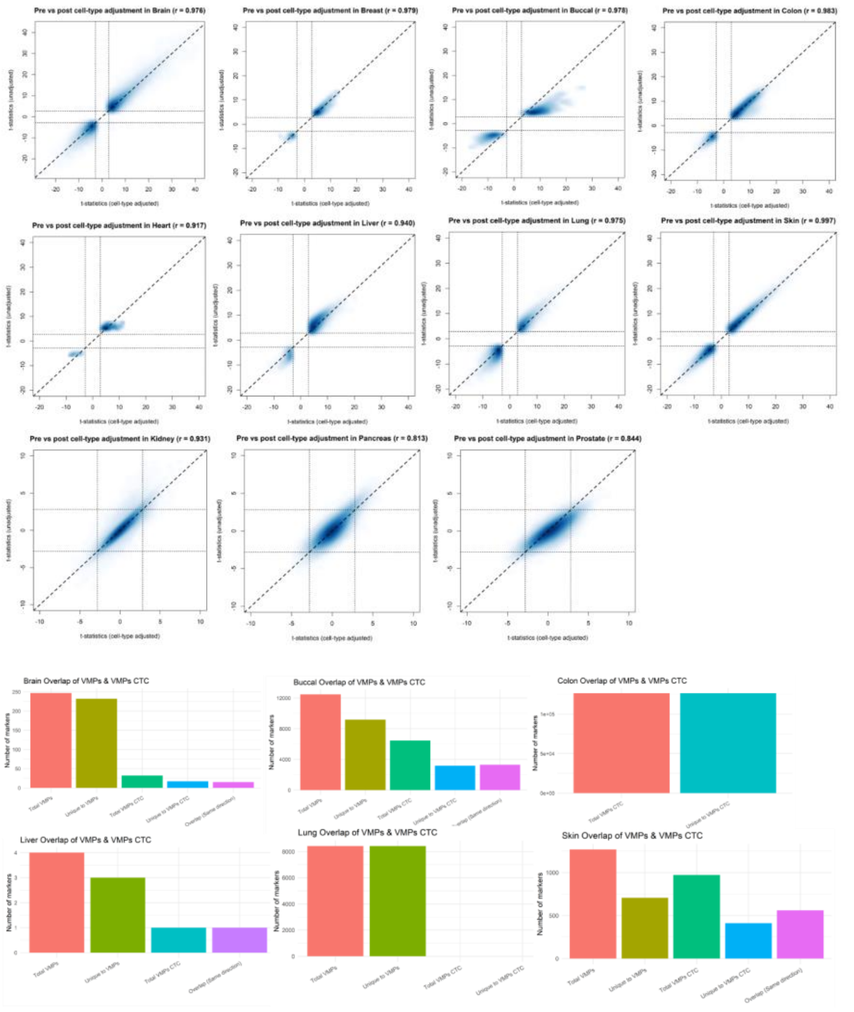

Supplementary Figure 2 Correlation plot between DMPs identifies with cell-type correction (x-axis) and without cell-type correction (y-axis) and number of VMPs identified before and after cell-type correction

Minor

1. The authors state ‘hemi-methylated’ (line 247) – which specifically refers to DNAm on only one of the two strands is methylated [3] – are the authors instead referring to intermediately methylated loci?

Response: We thank the reviewer for this point and apologise for the oversight. We have changed it to intermediately methylated for accuracy.

2. Pearson capitalised (line 739).

Response: We have changed capitalisation accordingly.

1. Oliva, M., et al., DNA methylation QTL mapping across diverse human tissues provides molecular links between genetic variation and complex traits. Nat Genet, 2023. 55(1): p. 112-122.
2. Mansell, G., et al., Guidance for DNA methylation studies: statistical insights from the Illumina EPIC array. BMC Genomics, 2019. 20(1): p. 366.
3. Sharif, J. and H. Koseki, Hemimethylation: DNA's lasting odd couple. Science, 2018. 359(6380): p. 1102-1103.

Response: We added the suggested references.

Reviewer #3 :

Overall, this is a statistically robust study which, by integrating many datasets, could serve as a reference to understand tissue specific methylation trajectories. As the authors write, there has been limited research into age-related methylation change outside of select tissues. Their study addresses this gap in the literature.

Major concerns

The major problem with this study is that it suffers from profound issues in the communication of findings, related to the text and figures. Textually, the manuscript is quite verbose and repetitive and should be greatly abbreviated. Additionally, many conclusions are interpreted as causal or relevant to aging as a whole, when they are purely correlative and relevant only to epigenetic aging specifically. The main weaknesses of the figures are that (1) they are not conducive to comparing the results across tissues, despite this being the main focus of the study, and (2) are poorly formatted in terms of the text size, legend, and color. So, while the methods and study design are sound, the presentation and communication of the results prevent this manuscript from being useful.

Response: We have made significant changes to the manuscript according to all reviewers' suggestions, including adding Figure, supplementary Table and format.

Specific points

1) A figure panel which makes it easy to compare the results across tissues is necessary for the presentation of the results in Figures 1, 2, 3, and 4. Suggestions for figures 2 and 4 follow, but this should be applied to all four of these figures. The study is meant to facilitate comparison of methylomic changes across tissues and so this is essential.

Response: We thank the reviewers' suggestions and have applied the changes accordingly.

2) Figure 2. The pie charts are aesthetically pleasing but not conducive to cross-tissue comparisons. The results from this figure would be clearer if presented in another form. I leave it to the authors to decide what that should be. One option would be a bar graph where tissues are arranged along the x-axis, each tissue has 3 bars whose height denotes the count of CpGs which begin lowly, intermediately, or highly methylated, and each of these bars is a "stacked" bar, where one color within the bar shows the proportion of CpGs which gain methylation and another color shows the proportion which lose methylation (within each starting-methylation class). In the new rendition of this figure, color should be used more thoughtfully to add interpretability instead of the current state where the colors chosen appear to be random, yet are re-used across panels.

Response: We thank the reviewers' suggestions and have applied the changes accordingly.

3) Figure 4. A figure which shows the effect size of entropy gain/loss in each tissue should be added to allow comparison across tissues. This could be a barplot where for each tissue there are 4 bars the height of each corresponds to the slope of entropy gain/loss for that class of CpG sites in that tissue. The statistical of this slope should be reported along with the effect size (in the figure).

Response: We thank the reviewers' suggestions and have applied the changes accordingly.

4) For DMPs and VMPs, results are presented across all datasets for a tissue. Why then for entropy are the results only from the largest dataset from each tissue? The analysis of the association between entropy and age should be re-done using all datasets for consistency.

Response: All available datasets for each tissue were included in the entropy analyses. Specifically, entropy was calculated within each dataset independently, and the association between entropy and age was tested separately per dataset before being combined across studies. Therefore, the statistical inference is based on all datasets, consistent with the DMP and VMP analyses. For presentation purposes only, we displayed the results from the largest dataset per tissue in the main figures. This was done for three main reasons. 1) To avoid overloading the manuscript with highly repetitive panels showing near-identical trends across datasets. 2) Provide the most statistically stable and visually representative example for each tissue (as the largest dataset typically has the narrowest confidence intervals and highest power). 3) Maintain consistency of visual scale and clarity in the figures. Importantly, the direction and magnitude of the entropy–age association were highly concordant across datasets within each tissue. The full multi-dataset results (including effect sizes and significance values for each individual dataset) are provided in the Supplementary Materials.

5) Why were skeletal muscle, adipose tissue, blood, and brain chosen for WCGNA? Did these tissues have the greatest number of samples? Either a quantitative metric should be used to justify the choice of which tissues to do WCGNA on (such as number of samples) or results from all tissues should be shown.
Response: Thank you for this important comment. WGCNA was performed independently in all tissues included in the study, not only in skeletal muscle, adipose tissue, blood, and brain. The four tissues shown in the main manuscript were selected for representation purposes to maintain clarity and readability, as the article is already extensive. These tissues were chosen because they represent biologically distinct systems (metabolic, immune, neuromuscular, and central nervous system), thereby providing a broad overview of ageing-associated network architecture across the body.

The selection was not based solely on sample size. Although these tissues are among the well-powered datasets, WGCNA was conducted in all tissues that passed quality control and network stability criteria. The analytical framework (network construction, module detection, and module–age association testing) was applied identically across tissues. While networks are built separately per tissue, the gene–gene interaction inference process is the same, allowing consistent cross-tissue comparison and a multi-tissue systems-level interpretation of ageing.

To ensure full transparency, results for all tissues are being made available through the accompanying webtool, (https://bioinformatics.erc.monash.edu/apps/human-aging-atlas/) where complete module statistics and associations can be explored. We have clarified this in the revised manuscript.

6) The authors conclude that "that aging is not solely characterized by stochastic drift" because they find age-related methylation changes which are (1) tissue-specific, (2) directional, and (3) are enriched in certain pathways. To make such a statement, the authors should show that a stochastic model cannot recover such patterns. Results from studies such as Myer et al. 2025 persuasively make the opposite claim. One could imagine that early in life, different tissues start with different methylomic patterns from which point stochastic drift accounts for all epigenetic change. Perhaps, this model could explain all

three observations mentioned above in which case aging would be solely characterized by stochastic drift. Generally, highly confident statements regarding the source or reason of methylomic change should be removed or supported with further analysis.

Response: We thank the reviewer for this thoughtful and important comment. We agree that stochastic drift likely contributes to age-associated methylation changes and that tissue-specific starting states could, in principle, generate divergent aging trajectories under a drift-based model. Our intention was not to exclude stochastic processes, but rather to highlight that the observed patterns are not fully consistent with a purely random model of methylation change.

Specifically, we observed directional methylation shifts across tissues, enrichment within defined biological pathways, and tissue-dependent entropy dynamics based on Shannon entropy analysis. While stochastic drift would predict a general increase in epigenetic entropy, entropy changes were not uniform across tissues, and many CpGs demonstrated coordinated, directional shifts rather than symmetric variance expansion. These findings suggest that, alongside stochastic processes, structured and potentially regulated epigenetic remodeling contributes to aging-associated methylation patterns.

To avoid overstatement, we have revised the manuscript (Page 14, lines 558-569) to clarify that our results are consistent with both stochastic and non-random components of epigenetic aging, rather than excluding drift as a contributing mechanism.

7) Throughout, the authors make overly strong statements based on purely correlative analyses. For example, “Key disruptors, including PCDHGA1, MEST, HDAC4, and HOX genes, exacerbated aging signals across tissues.” These genes are not causally validated "disruptors of aging signals" they simply changed the WCGNA clustering when simulated to have different methylation values. Relatedly, the terminology of "driver gene," used in Figure 5 and onward, should be changed because "drive" implies causation, of which none has been shown. Numerous statements such as this should be clarified and toned-down.

Response: We thank the reviewer for this important clarification. We fully agree that our analyses are correlative and do not establish causation. Our use of terms such as “driver gene” and “key disruptor” was intended in the network-analytic sense commonly used in WGCNA and gene–gene interaction frameworks, where these terms refer to highly connected or influential nodes whose perturbation alters module structure or eigengene behavior. In our simulations, these genes were identified as central network nodes whose in silico methylation perturbation led to measurable shifts in module activity, indicating network sensitivity rather than causal validation of aging mechanisms.

To avoid implying causation, we have revised the manuscript throughout to use more precise terminology, including “network-central genes,” “module-influential genes,” or “high-connectivity nodes,” and have clarified that these findings reflect network topology and in silico perturbation effects rather than experimentally validated causal drivers of aging. We have also softened related language to explicitly state that these results generate hypotheses regarding regulatory importance rather than demonstrating direct biological causation.

8) Many statements imply that the results of this study reflect characteristics of aging as a whole. E.g., “Additionally, using advanced computational tools, we have identified key drivers of ageing in different tissues and uncovered unique ageing pathways and coordinated methylation patterns among tissues." This is inaccurate. This study only looked

at *epigenetic aging* which may or may not relate to other aspects of aging writ large. This should be clarified throughout.

Response: We have revised the manuscript throughout to explicitly refer to “epigenetic aging” or “age-associated DNA methylation changes” rather than aging more broadly. Statements that could be interpreted as generalizing to aging writ large have been refined to ensure conceptual precision. We believe these revisions more accurately reflect the scope of our analyses and prevent overextension of the findings.

9) The text is very long and repetitive. For example, DMP, VMP, and entropy are defined and explained multiple times. The authors should consider how they can massively streamline the manuscript. Suggestions:

Response: We have adjusted the manuscript where relevant. Please see detailed comments for each suggestion below.

10) The first results section can be almost entirely eliminated without loss of information.

Response: Thank you for this suggestion. We respectfully disagree. This introductory Results section was intentionally structured to provide a clear conceptual and methodological foundation for the study. During earlier consultations and feedback from experts in epigenetics and ageing research, we were specifically encouraged to clearly define and justify the three complementary analytical layers (DMPs, VMPs, and entropy) before presenting downstream comparative and network-level results. Given that variance-based and entropy-based methylation analyses are less commonly applied than traditional DMP analyses, this clarification was considered particularly important to ensure interpretability and accessibility for a broad readership. The section also establishes the scale, rigour, and harmonisation framework of the atlas (17 tissues, >15,000 samples, 131 datasets), which is central to understanding the robustness of the subsequent cross-tissue meta-analyses. Removing it would reduce clarity regarding how the multidimensional ageing framework was constructed and why these complementary metrics were used. That said, we have carefully revised the section to improve conciseness, reduce redundancy, and streamline descriptions while preserving the key information necessary for proper interpretation of the study.

11) Figure captions can be made shorter and more precise

Response: We appreciate the reviewer’s suggestion. Given the complexity of the multi-tissue analyses and the methodological steps involved, we have intentionally retained a higher level of detail in the captions to ensure that each figure can be interpreted independently of the main text. We believe this improves readability and accessibility for readers navigating specific results without repeatedly cross-referencing the Methods section.

Where possible, we have streamlined wording and removed redundancy while maintaining sufficient detail to support accurate interpretation.

12) The discussion is far too long and should be shortened to more succinctly and elegantly integrate and discuss the findings of the study. For example, "Recent research links hypermethylation in the PCDHG family to reduced white matter in the brain, a marker of accelerated cognitive decline. This connection is particularly intriguing since previous studies have shown that abdominal fat can predict decreases in cerebral white matter" how is the fact that abdominal fat predicts decreases in cerebral white matter related whatsoever to the

findings of this study? There are also multiple epically run-on sentences such as from line 530-535.

Response: We have taken the reviewers' suggestion on board and substantially revised the discussion and it is now significantly shorter, precise and direct.

13) The figure legends, axes tick labels, axes labels, and much of the text in figures is too small and low-resolution to read. Nearly every panel of every figure must be re-formatted to achieve legibility. Colors should be used more thoughtfully (e.g., diverging color maps should not be used for monotonic data).

Response: Figures have been improved and changed according to reviewers' suggestions.

14) The information in Figure 3 would better be represented as a volcano plot, where the x-axis is the coefficient relating age and the squared residual from `$\epsilon i2= \gamma 0 + \gamma 1 \cdot Age + \delta$` and the y-axis is the log10 p value for this coefficient.

Response: We thank the reviewer for the suggestion. We agree that a volcano plot is a useful way to summarise the association between age and variability by jointly displaying effect size (γ1) and statistical significance. However, the aim of Figure 3 is to visualise the underlying VMP phenomenon (i.e., age-associated changes in methylation variability) rather than only to rank loci by significance. For this reason, we believe the current scatter-based representation better conveys the biological interpretation of VMPs (changes in variability rather than mean shifts).

To improve interpretation, we have adjusted the figure to match panels and scales.

15) It is unclear which, and how many, covariates were adjusted for in the DMP and VMP analyses. A list of the covariates adjusted for in each study should be presented. If the same set of covariates was not adjusted for across all studies, the authors should justify this inconsistency or use a set of covariates available in all studies.

Response: Covariate selection was performed at the level of each individual study rather than imposing a single uniform set across all datasets. This strategy was adopted because the included studies differ substantially in tissue type, study design, platform, and availability of phenotypic and technical metadata. Restricting analyses to only covariates shared across all studies would have resulted in omission of important study-specific confounders and reduced the validity of within-study effect estimates.

Accordingly, for each dataset, age-association models for both DMP and VMP analyses included all relevant biological and technical covariates available for that study (e.g., sex, batch, cell composition estimates, and other cohort-specific factors where applicable). These models were fitted independently within each study prior to integration. The full list of covariates adjusted for in each dataset is provided in Supplementary Table 1, which we now explicitly reference in the Methods (line 683).

This study-level modelling approach, followed by summary statistic–based meta-analysis, reduces heterogeneity arising from unmeasured confounding while allowing optimal adjustment within each cohort.

16) It is unclear to me what Figure 5B/D/F/H show. How is a gene determined to be a "top gene driving enrichment in age-associated modules"? The methods section merely says "Modules ... top hub CpGs and driver genes were extracted" but does not specify how this was done. What metric was used to identify driver genes? How was statistical significance

assessed? A statistical significance cut-off with multiple testing correction should be used to ensure the “driver genes” are meaningful. This is especially important because of the authors’ focus on the frequency of PCDHG genes being found to be "driver genes” across tissues.

Response: We agree that the original description was insufficiently explicit and have revised the Methods (page 20, lines 808 to 819) and figure legends accordingly.

In this study, “driver genes” were not defined based on differential methylation significance testing alone, but rather using a network-based prioritisation framework. Specifically, driver genes were identified as genes with high intramodular connectivity (hub genes) within age-associated WGCNA modules, based on module membership (kME) and gene significance for age. These metrics capture the extent to which a gene is both central to a co-methylation module and strongly associated with the aging phenotype.

For each age-associated module, genes were ranked by their combined connectivity and age association metrics, and the top-ranked genes were designated as driver genes. Statistical significance of age association was assessed at the CpG level during module construction with multiple testing correction, while driver gene designation reflects network centrality rather than an additional hypothesis test. We therefore interpret driver genes as network-prioritised candidates rather than definitive causal regulators.

Figures 5B/D/F/H display the frequency with which these top-ranked driver genes recur across age-associated modules and tissues, highlighting reproducible network-central genes such as members of the PCDHG family. We have clarified this rationale, added explicit definitions of driver gene metrics, and revised the figure legends to improve interpretability.

References

1 Teschendorff, A. E., Zhu, T., Breeze, C. E. & Beck, S. EPISCORE: cell type deconvolution of bulk tissue DNA methylomes from single-cell RNA-Seq data. *Genome Biol* **21**, 221 (2020). https://doi.org/10.1186/s13059-020-02126-9

2 Zheng, S. C. *et al.* EpiDISH web server: Epigenetic Dissection of Intra-Sample-Heterogeneity with online GUI. *Bioinformatics* **36**, 1950–1951 (2019). https://doi.org/10.1093/bioinformatics/btz833

3 Zheng, S. C. *et al.* Correcting for cell-type heterogeneity in epigenome-wide association studies: revisiting previous analyses. *Nat Methods* **14**, 216–217 (2017). https://doi.org/10.1038/nmeth.4187

Dear Editor,

We thank you and the reviewers for taking the time to review this manuscript. We agree with the editor that this should be published as “Analyses”.

We have now responded to the minor concerns raised by the reviewers and revised the manuscript accordingly.

Specifically, as requested, we have softened and/or included an explanation for the context of 'global disruptor genes' (in the absence of causal evidence), and we resubmitted the Figures in high resolution. We have also added an additional supplementary Table in response to Reviewer’s #3 comment #2.

Nir Eynon, on-behalf of the co-author.

Reviewer #1:

The authors have submitted a substantially revised manuscript, alongside a very thorough and thoughtful rebuttal. Constructing an epigenetic aging atlas across 17 human tissues utilizing over 15,000 methylomes is a massive undertaking, and this manuscript provides a highly valuable resource to the aging research community.

I commend the authors for their diligent efforts in addressing the concerns raised by all reviewers during the first round. The revisions have significantly elevated the statistical rigor, clarity, and interpretability of the study.

Response: Thank you for this positive comment.

Minor points:

1. Terminology Consistency: While the authors successfully removed "driver genes," the term "global disruptor" is still utilized in the main text (e.g., Line 419: "a limited set of 'global disruptor' genes"). While acceptable within the context of the in silico robustness simulations, I recommend adding a brief, one-sentence inline clarification when the term is first introduced in the main text to remind readers that this refers strictly to network topology and simulated mathematical disruption, not a biologically validated causal disruption.

Response: Thank you for the suggestion. We have now added a sentence clarifying this in the text.

2. Figure Presentation and Legends: Please further optimize the aesthetic layout of the figures and refine the accompanying legends. Currently, the captions for the Supplementary Figures appear somewhat sparse. Expanding these legends to be more comprehensive, consistent, and standardized will greatly assist readers in fully grasping the complex visual information without needing to constantly cross-reference the main text.

Response: We have now submitted high-resolution figures and adjusted the captions to include more details as suggested.

3. Supplementary Callouts: please check the supplementary callouts in the text. For instance, on Line 421, 425 and 434, the text references "(Supplementary Table 7)" three times in close succession regarding module disruption; ensure the referencing flows naturally.

Response: We have now removed repetition on supplementary tables within same subsection.

Overall, this is an excellent, rigorous, and highly comprehensive study. The authors have fully satisfied the requirements of the peer review process.

Response: Thank you for this positive comment.

Reviewer #2:

Reviewer #2 has indicated that we responded to all their previous comment, and they are satisfied with the revisions previously made. We thank the reviewer for all previous suggestions and there were no further comments from them.

Reviewer #3:

The authors have submitted an improved manuscript and addressed many of our suggestions. However they continue to overstate the conclusions that can be made as a result of their study, in particular the implications of their findings on the relevance of stochastic drift to epigenetic aging.

1. The authors did not make any attempt to address our point #3, barely changing the original figure. They should re-attempt to address our original comment as a figure that allows easy comparison of entropic change across tissues is of utmost importance for conveying the results. The change the authors made to Figure 2 was very nice – something similar here would be helpful. Original comment: " Figure 4. A figure which shows the effect size of entropy gain/loss in each tissue should be added to allow comparison across tissues. This could be a barplot where for each tissue there are 4 bars the height of each corresponds to the slope of entropy gain/loss for that class of CpG sites in that tissue. The statistical of this slope should be reported along with the effect size (in the figure)."

Response: We have now added to the entropy plots the (slope/year) change observed in each case. We have adjusted to the same scale and respectfully disagree with the suggestion of barplots. These have become very convoluted while providing exactly the same information as the current plots after we have added the slope change to each tissue.

2. The authors have inadequately addressed our previous comment (#6) regarding entropic/stochastic epigenetic drift during aging. They write "The predominance of age-associated hypermethylation across multiple tissues challenges the prevailing notion that aging primarily reflects stochastic drift toward intermediate methylation states." This is not an accurate conclusion from the data. As they nicely present, the majority of tissues begin in a state of hypomethylation (Figure 2). Thus, if every CpG merely drifted towards an intermediate methylation state, age-associated hypermethylation is exactly what would be expected. They also argue that "directional methylation shifts across tissues,

enrichment within defined biological pathways, and tissue-dependent entropy dynamics based on Shannon entropy analysis" rebut the idea of stochastic drift. This is false. Each of these findings are plausible under pure stochstic drift assuming each tissue & biological pathway has a different starting methylation pattern. To amend this, the authors should significantly revise all portions of the manuscript which address the idea of stochasticity in epigenetic aging. If they want to contribute to this important area of research they should conduct a more nuanced analysis. Reflection upon these studies may assist the authors in this endeavor: Myer et al. 2025, Tarkhov et al. 2024, Tong et al. 2024.

Response: We agree with the reviewer that the predominance of hypermethylation cannot, by itself, be interpreted as evidence against stochastic drift, particularly in tissues that begin from predominantly low methylation states in younger individuals. We have now added an additional supplementary table, which should be used in conjunction with Figure 2 for correct interpretation. We have therefore revised the manuscript to remove statements implying that our data refute stochastic drift and instead interpret the findings using methylation-state transition analyses that distinguish direction of change from movement toward intermediate methylation levels.

3. The authors did not fully address our comment #7. The sentence we quote there – which wildly overstates their findings – is still present in the abstract. The authors should choose a more causally neutral term than "disruptor" there and throughout the manuscript.

Response: Thank you for the suggestion. We have now added a sentence clarifying this in the text, in line with the same suggestion by reviewer #1.

4. While some figures have been improved, figures 4-6 have highly illegible text (We tested this in 2 pdf viewers). This may be amended by increased text size, reduced figure complexity, or increased resolution.

Response: We have now submitted high-resolution figures and adjusted the captions to include more details as suggested.
